# Progress in organic photovoltaics for indoor application

**DOI:** 10.1039/d3ra02599c

**Published:** 2023-10-31

**Authors:** Swarup Biswas, Yongju Lee, Hyojeong Choi, Hyeong Won Lee, Hyeok Kim

**Affiliations:** a School of Electrical and Computer Engineering, Center for Smart Sensor System of Seoul (CS4), University of Seoul 163 Seoulsiripdaero, Dongdaemun-gu Seoul 02504 Republic of Korea hyeok.kim@uos.ac.kr +82-2-6490-2314 +82-2-6490-2354; b Central Business, SENSOMEDI 45, Yangcheong 4-gil, Ochang-eup, Cheongwon-gu Cheongju-si 28116 Republic of Korea; c Institute of Sensor System, SENSOMEDI, Seoul Biohub 117-3, Hoegi-ro, Dongdaemun-gu Seoul 02455 Republic of Korea; d Energy Flex Sagajeong-ro 65, Dongdaemun-gu Seoul 02553 Republic of Korea

## Abstract

Organic photovoltaics (OPVs) have recently emerged as feasible alternatives for indoor light harvesting because of their variable optical absorption, high absorption coefficients, and low leakage currents under low lighting circumstances. Extensive research has been performed over the last decade in the quest for highly efficient, ecologically stable, and economically feasible indoor organic photovoltaics (IOPVs). This research covers a wide range of topics, including the development of new donor–acceptor materials, interlayers (such as electron and hole transport layers), energy loss reduction, open-circuit voltage enhancement *via* material and device engineering, and device architecture optimization. The maximum power conversion efficiency (PCE) of IOPVs has already topped 35% as a consequence of these collaborative efforts. However, further research is needed to improve numerous elements, such as manufacturing costs and device longevity. IOPVs must preserve at least 80% of their initial PCE for more than a decade in order to compete with traditional batteries used in internet of things devices. A thorough examination of this issue is urgently required. We intend to present an overview of recent developments in the evolution of IOPVs.

## Abstract 2

Numerous potential uses for indoor organic photovoltaic cells.

## Graphical abstract

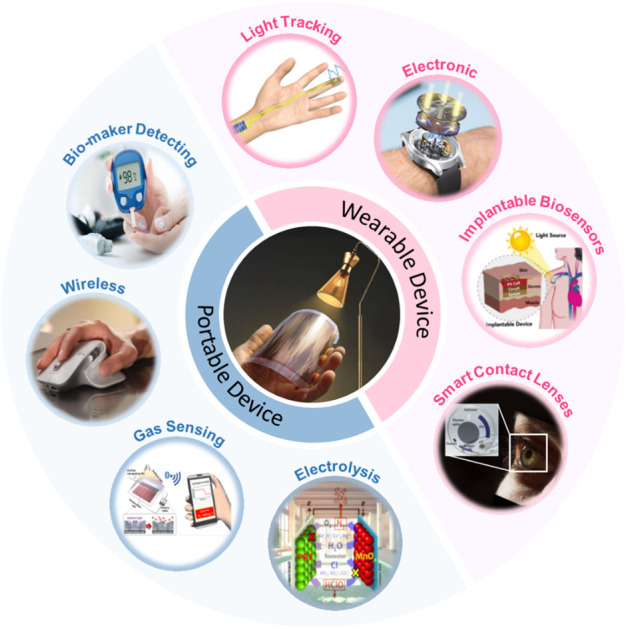

## 1. Introduction

The Internet of Things (IoT), which requires a multitude of small low-powered electronic devices like sensors, actuators, smart meters, and healthcare monitoring system devices, has resulted in a recent spike in demand for effective ambient-energy harvesting systems.^1–10^ In general, these devices are operated by extracting power from normal batteries, but these types of power systems need regular replacement and maintenance, which is very difficult because of the tinny and wireless nature of those devices.^11,12^ Consequently, harvesting of ambient energy is the most appropriate alternative solution. Most components of IoT networks operate indoors.^13,14^ Artificial lighting sources such as fluorescent lamps (FL), compact fluorescent lamps (CFL), halogen lamps, light-emitting diodes (LEDs) (both warm and cold white), incandescent lamps, and natural sunlight through windows are the most reliable and accessible resource for collecting energy and charging such devices.^15^ A photovoltaic (PV) device is the main tool needed for harvesting light energy. When compared to outdoor conditions, the light intensity is noticeably lower in indoor.^16^ The intensity of light has a significant impact on a variety of characteristics and performance parameters, including short-circuit current density (*J*_SC_), open-circuit voltage (*V*_OC_), fill factor (FF), and power conversion efficiency (PCE) of a PV device.^17^ The effect of light intensity on these factors may vary depending on the individual PV technology under consideration. For instance, *J*_SC_ of a PV device generally increases with light intensity. This is because higher light intensity results in more photons being absorbed by the semiconductor material, leading to a greater number of electron–hole pairs being generated and contributing to a higher current. Whereas, *V*_OC_ is relatively unaffected by changes in light intensity. However, under very low light conditions, the *V*_OC_ might show a slight increase due to reduced recombination losses. The FF, denoting the efficiency of converting incident light into electrical power within a solar cell, may enhance with escalating light intensity, owing to diminished recombination losses. Yet, under exceedingly high light intensities, the FF could potentially decline due to the PV device's non-ideal behaviour. Given the interdependence of these parameters on light intensity, the PCE of the device also exhibits reliance on light intensity. Among the several PV devices available, the organic photovoltaic (OPV) device stands out as an excellent alternative for harvesting indoor light. This preference stems mostly from its notable absorption coefficient in the visible spectrum (which corresponds to the irradiance spectra of common interior light sources). Furthermore, its tuneable optical characteristics provide an advantage in circumstances when the indoor illumination spectrum is well defined, potentially leading to increased efficiency. The intrinsic versatility of OPV technology adds to its allure, allowing for creative integration into a variety of surfaces such as tiny electronic devices, windows, walls, and even fabrics. This versatility opens the door to novel design concepts and aesthetic possibilities that would be impossible to realize with rigid solar technology. Furthermore, the cost-effectiveness of OPV production acts as an additional incentive for its use.^16,18–35^ On the other hand, when it comes to outdoor use, OPVs have various disadvantages. These include lower efficiency due to their restricted absorption band, which limits their practicality for energy-intensive outdoor scenarios that rely on optimizing energy output. Furthermore, the stability of OPV materials is weakened under outdoor condition that expose them to more demanding factors such as UV radiation, temperature variations, and moisture exposure. This exposure increases the likelihood of rapid deterioration and shorter operating lifespans.

Over the last decade, considerable advances have been achieved in the development of highly efficient and environmentally robust indoor organic photovoltaic (IOPV) devices, which have already exceeded 35% PCE.^36–38^ To achieve this objective, a range of approaches have been put into practice. Among these strategies, producing broad band-gap organic semiconductors with extraordinarily high absorption coefficients has received special attention. This focus is due to the fact that the emission spectra of many indoor light sources are mostly in the visible spectrum (400–700 nm). As a result, the optical bandgap of the semiconductors used in IOPV must lie within the energy range of 1.8–2.1 eV.^39,40^ In this context, an array of donor and acceptor materials suitable for conditions of low light irradiance have been synthesized.^41–43^ The greater energy barrier in between electrode and the active material within an OPV restricts charge carrier movement from the active layer towards the electrode. This may result in poor performance of the device during its operation indoors. To address these issues, many semiconducting (both inorganic and organic) materials were employed as interlayers (*i.e.*, hole transport layer (HTL) and electron transport layer (ETL)) in between electrode and active layer of IOPVs.^36,44–48^ Transparent conducting electrodes (TCEs) based on a range of materials have indeed been produced in relation to numerous semiconducting interlayers. Because the parasitic resistances of the device are partially reliant on the surface morphology, conductivity, and structural flaws of the TCEs, TCEs play an important role in improving the performance of IOPVs.^49–54^ Finally, since the photon absorption abilities of various layers (particularly the active layer) are strongly dependent upon their thickness, the performance of a OPV may be modified by optimizing the thickness values of different layers of the device. As a result, several research have been carried out in the recent few years to improve the design of IOPVs utilizing optical simulation using the finite-difference time-domain (FDTD)-based modeling approach.^55–65^ A detailed review reveals that, although we have achieved over 35% PCE from an IOPVs *via* extensive research,^38,66^ the commercialization of IOPVs has not yet been achieved because of several drawbacks such as shorter lifespan and lower stability. Therefore, a detailed review of this topic will be very helpful to identify the challenges that must be addressed in the near future.

In this extensive review, we deeply examine a range of methodologies utilized in the development of highly efficient IOPVs. Our investigation includes a detailed analysis of various photovoltaic components used in IOPVs' active layer. We also investigate the incorporation of different donor–acceptor semiconductors into this active layer, as well as the evolution of many interlayers designed for the best IOPV performance. We also extensively investigate the application of optical simulation techniques to enhance device architecture. Concluding our study, we provide a comprehensive discourse on the commercial potentials of IOPVs, coupled with insights into their promising future prospects.

## 2. Various indoor light sources

**Fig. 1:**
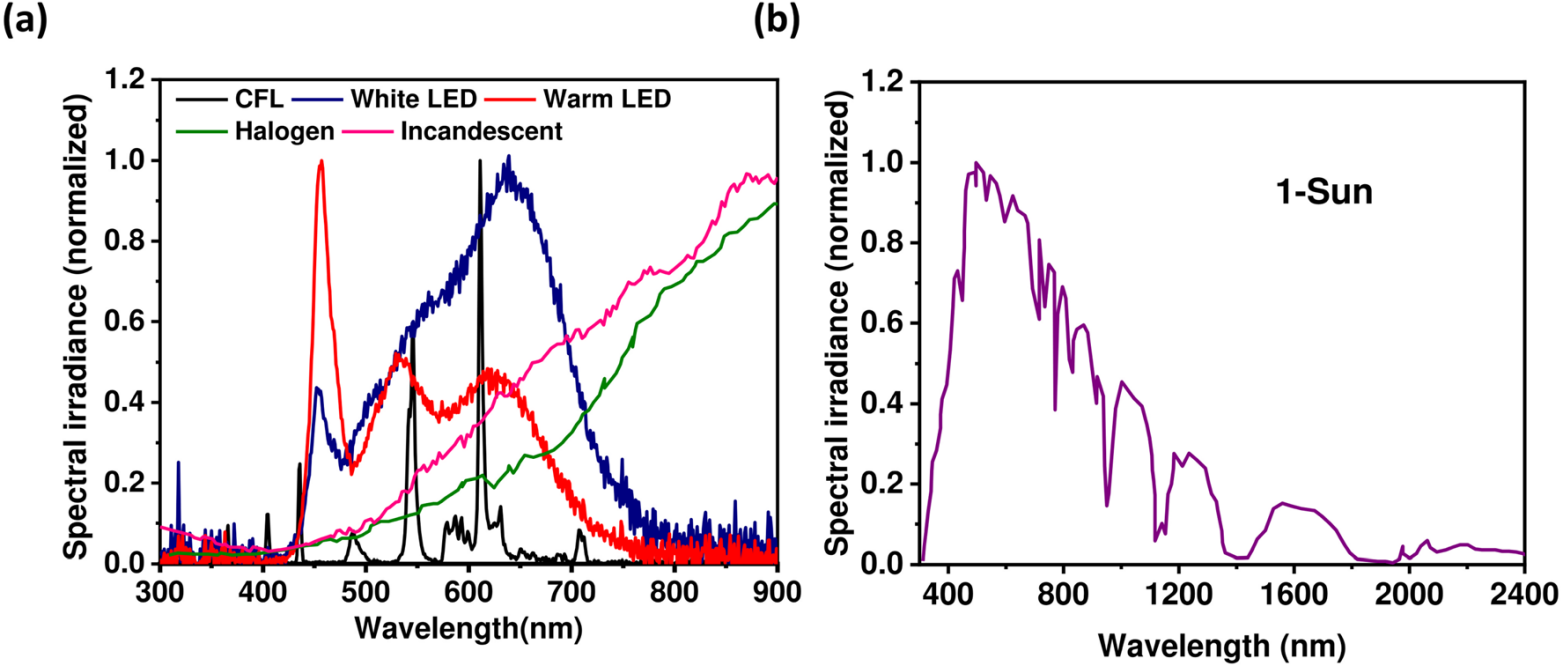
(a) Irradiance spectra of different indoor light sources;^16^ (b) solar spectra (AM1.5G).^16^

The light source is an essential component of an indoor light energy harvesting system. We have already mentioned that various types of light sources are used as major illuminators of indoor environment. These light sources differ in their wavelength ranges and the intensity of their irradiance spectra, allowing them to be distinguished from one another.^16^ As depicted in Fig. 1a, the irradiance spectrum of the CFL tube light reveals notable peaks around ∼400 nm, ∼450 nm, ∼550 nm, and ∼600 nm, while the cold white LED light exhibits pronounced peaks at approximately ∼450 nm and ∼550 nm. In contrast, the irradiance spectra of halogen and incandescent bulbs display a gradual and consistent increase across the wavelength span of 300–900 nm. Additionally, the wavelength distribution of light emitted by these distinct sources varies. Notably, the LED light source demonstrates a broader and more continuous dispersion of energy compared to the CFL. Moreover, there are distinctions in the light quality generated by the different sources; they produce light with differing irradiance power intensities while maintaining the same luminance value. Conversely, natural sunlight exhibits a broad range (wavelength) of irradiance spectra (Fig. 1b). As a consequence, OPVs typically demonstrate distinct spectral responses to various light sources when held at a constant luminance value. This phenomenon gives rise to varying levels of harvested light energy by a PV panel, depending on the specific artificial light source it operates under at a fixed luminance value. Consequently, the creation of customized OPVs tailored for indoor usage presents a challenging endeavor. The optimization of diverse OPV parameters is imperative to accommodate indoor lighting conditions. The device's design must be adept at achieving efficient operation under the prevalent illumination of frequently employed indoor light sources. Recent years have witnessed a concerted effort from researchers to optimize numerous device parameters for indoor applications. Additionally, the pursuit of synergistic semiconductor materials—characterized by broader absorption spectra that align well with the irradiance spectra of commonly utilized artificial light sources—has emerged as a central objective in the quest to produce exceptionally efficient OPVs tailored for indoor.

## 3. Performance measurement of OPVs under indoor light sources

**Fig. 2:**
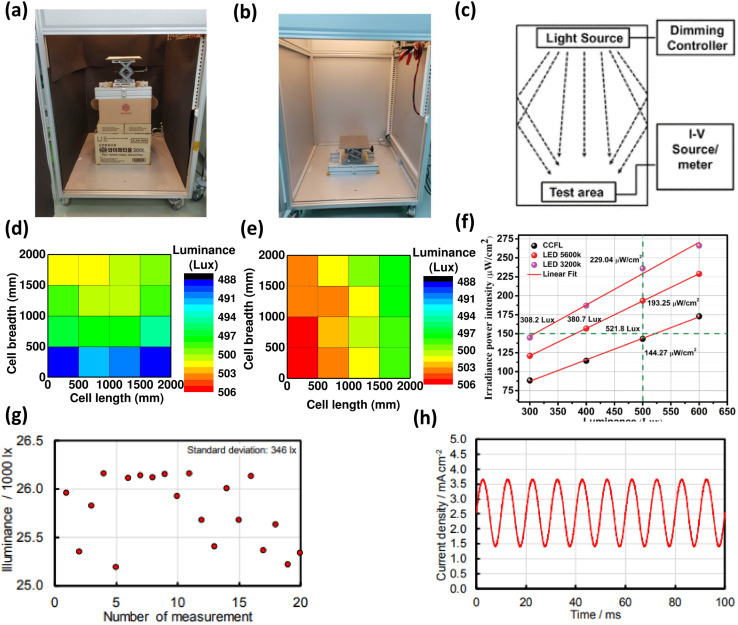
Experimental setup for low irradiance measuring unit: (a) shorter distance between light source (CFL) and measuring unit, (b) longer distance (1.4 m) between light source (CFL) and measuring unit, and (c) schematic at standardized condition, variation of luminance value of incident light on different position of PV cell for both (d) short, and (e) long-distance between light source (CFL) and measuring unit, (f) relation between irradiance power intensity and luminance of different sources of indoor light; [reproduced with permission,^67^ copyright 2020, IEEE]. (g) Effect of the exposure time on illuminance measurement results repeated 20 times determined for a lighting apparatus (ALPHAX Co., Ltd Type AL-50FL): number of integrations was set to 5 (exposure time was set to 12.8 ms in the automatic mode), (h) current density fluctuation of c-Si PV (power generation area: 1.96 cm^2^) caused by light blinking of a lighting apparatus (ALPHAX Co., Ltd Type AL-50FL); [reproduced with permission,^68^ copyright 2018, The Electrochemical Society of Japan].

It is evident that regular testing of OPVs is imperative. Hence, creating a simulated optimal testing environment for indoor light exposure is crucial for obtaining precise outcomes. It's important to acknowledge a fundamental distinction between outdoor and indoor lighting conditions. Unlike outdoor condition where PV devices directly receive sunlight, indoor environments frequently lack direct sunlight. Consequently, the significance of diffused light, resulting from reflection off various surfaces, becomes heightened as it contributes to powering indoor PVs (Fig. 2a–c).^67^

To establish a suitable testing environment for PV devices during experimental trials, the mitigation of uneven light distribution holds paramount importance. In one word standardization of test configuration for developing realistic indoor light conditions is highly needed. In this context, recent work by Kim *et al.* focused on refining the measurement system by optimizing the spacing between the light source and the device platform.^67^ Their objective was to establish an optimal indoor lighting setup. The study's outcomes underscore that fine-tuning the distance between the light source and the test device can yield the desired diffused light effect (Fig. 2d and e).^67^ Notably, the researchers identified that achieving a more accurate assessment of the PV device's performance variation under different light sources is attainable by considering a constant irradiance power intensity value for the light sources, as opposed to a fixed luminance value. This approach is particularly insightful since diverse indoor light sources exhibit varying irradiance power intensity values for a given luminance level (Fig. 2f).

In contrast, Saito *et al.* noted that readily available indoor light sources in the market can experience fluctuations in luminance when powered by an AC source (Fig. 2g).^68^ This variability has the potential to introduce fluctuations in the results of PV device tests. To address this issue, the researchers introduced a method aimed at mitigating the impact of these luminance fluctuations on the *J*–*V* measurements of PV devices (Fig. 2h).^68^ Furthermore, their study involved the creation of an LED light irradiation apparatus, designed to enhance the dependability and reproducibility of *J*–*V* measurements for PVs. This equipment demonstrated remarkable temporal stability in illuminance and uniformity across the test plane, fulfilling the rigorous requirements set forth by the Class A solar simulator standards prescribed in IEC 60904-9.

In 2021, Hou and colleagues introduced a refined measurement procedure to enhance the precision of characterizing OPVs under indoor lighting conditions.^69^ Their study involved a comprehensive series of experiments aimed at dissecting the sources of errors encountered in indoor PV measurements. The researchers effectively demonstrated that the temporal stabilities and homogeneity factor of commonly utilized LED and FL light sources can yield satisfactory outcomes for PV measurements, provided both factors are rigorously assessed prior to conducting measurements. To achieve accurate results, they emphasized the necessity of calibrating spectral irradiance and light intensities using a precise spectrometer, while discouraging the utilization of lux meters. The research encompassed an evaluation of cell area and the area ratio of the aperture to the cell. Consequently, they recommended employing cells of 1 cm^2^ or larger, accompanied by slightly smaller apertures, for optimal PV measurements. Furthermore, the researchers stressed the meticulous elimination of stray light effects, encompassing light reflection, and scattering caused by measurement apparatus, to minimize potential interference in PV measurements.^69^

The PCE of a PV device can be estimated by utilizing the following equation (eqn (1))^69^1
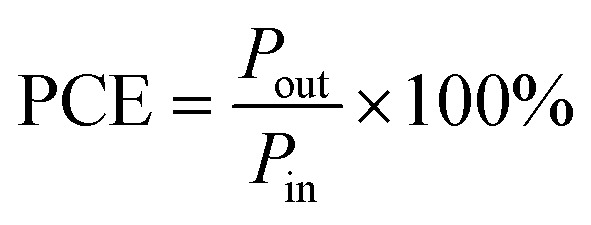
where *P*_out_ is the output power which can be estimated by the following equation (eqn (2))^69^

2 *P*_out_ = *V*_OC_ × *J*_SC_ × FF

and *P*_in_ is the power intensity of incident light, which can be estimated by eqn (3),^69^3
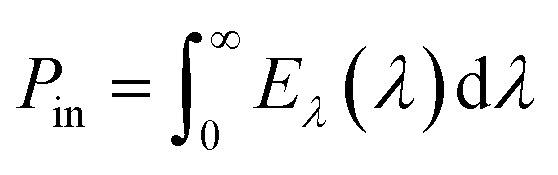
where *E*_*λ*_ is spectral irradiance (W m^−2^ nm^−1^), and *λ* is the wavelength.

The value of *J*_SC_ can be achieved in two ways, one is from *J*–*V* characteristics curves and another is by calculating from the external quantum efficiency curve (EQE(*λ*)) (eqn (4)). According to eqn (4), the short circuit current density (*J*_SC,EQE_) calculated from EQE(*λ*) can be expressed as,^69^4
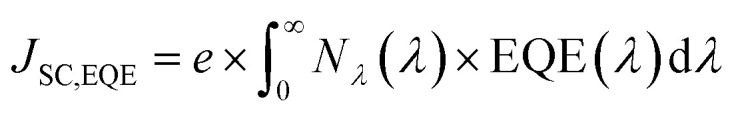
where (*N*_*λ*_(*λ*) = *E*_*λ*_ × *λ*/*hc*, considering *E*(*λ*) as energy associated to a particular *λ*, *h* is Planck constant and *c* is the speed of light) is the photon numbers per nanometre per square centimetre. It is evident from eqn (1)–(3) that precise estimation of *J*_SC_ is crucial for accurately determining the PCE value of a PV device under indoor lighting conditions. When determining *J*_SC_ from the measurement of *J*–*V* characteristic curves, the estimation of *J*_SC_ can be influenced by several factors such as leakage current, contact losses, non-ideal diode behaviour, measurement artefacts *etc.* potentially leading to both overestimation and underestimation of *J*_SC_. This may results estimation of inaccurate device PCE. Therefore, Hou *et al.* has recommended that *J*_SC,EQE_ of the OPV is essential for evaluating the reliability for the *J*_SC_ obtained from the PV measurement. Therefore, the EQE(*λ*) curves of the cells and the spectral characteristics of the incident lights must be provided and also the deviations between *J*_SC_ and *J*_SC,EQE_ should be low, *e.g.*, <5%.^69^

The comprehensive review reveals that while establishing a standardized protocol for assessing the performance of OPVs under indoor lighting is crucial for precise determination of device performance metrics, only a limited number of endeavours have been undertaken thus far. A concentrated emphasis on addressing this specific area is greatly warranted in the coming times.

## 4. Various donor and acceptor materials for IOPVs

**Fig. 3:**
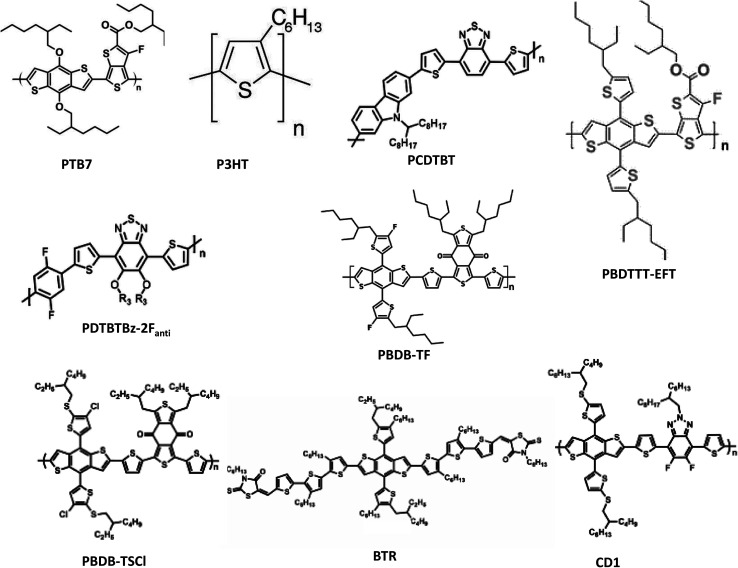
The chemical structures of numerous donor materials utilized in indoor OSCs.

**Fig. 4:**
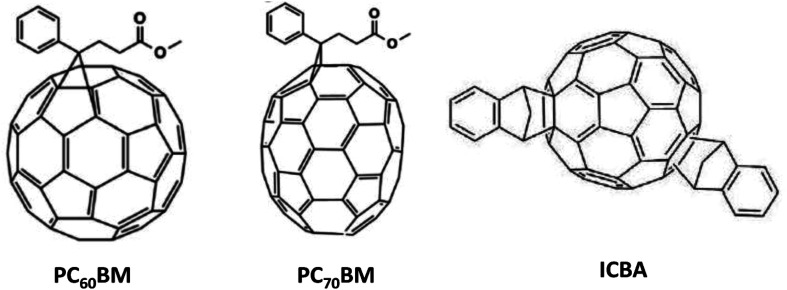
The chemical structures of several fullerene-acceptor materials utilized in indoor OPVs.

**Fig. 5:**
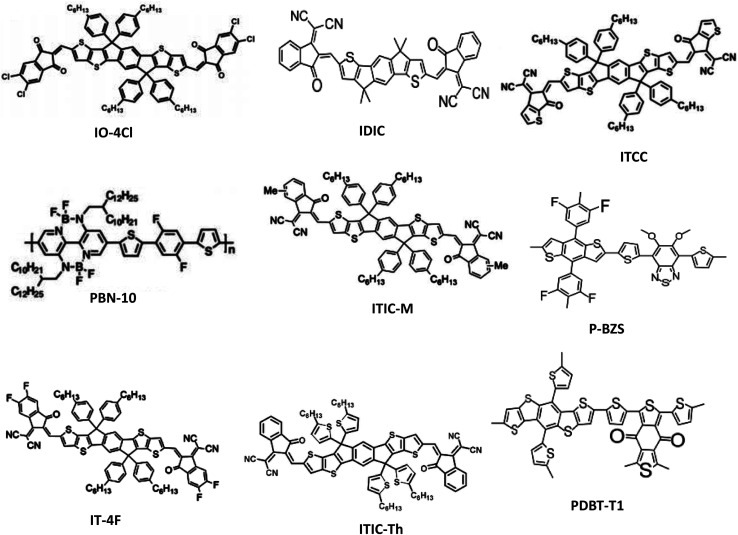
The chemical structures of different non-fullerene-acceptor materials utilized in indoor OPVs.

Distinct operational processes between indoor and outdoor OPV cells arise primarily from disparities in light intensity, spectral composition, and environmental variables. In the case of outdoor OPVs—integrated into diverse exterior surfaces like building facades, windows, and wearables to produce renewable energy for an array of applications—the light source comprises natural sunlight, characterized by its wide spectrum and heightened intensity in contrast to indoor illumination. Therefore, the selection of specific active materials (donors and acceptors) becomes crucial to ensure absorption across a broader wavelength range. Moreover, the design of outdoor cells necessitates the incorporation of highly durable materials capable of withstanding the rigors of an exposed environment. Conversely, IOPVs, apt for low-power uses like energizing compact electronics, sensors, or energy-efficient lighting in indoor settings, predominantly function within the realm of artificial illumination—examples being LEDs, FLs, and incandescent bulbs. This artificial light possesses lesser intensity in comparison to natural sunlight. Thus, the judicious selection of active materials with a narrower absorption band and elevated absorption coefficients becomes pivotal in the crafting of notably efficient IOPVs. To develop a suitable active material for highly efficient IOPVs, appropriate donor and acceptor materials must be developed. Several factors must be considered for this purpose, including good spectral matching in between active layer's absorption spectra and the light's illuminance spectra, adjusting the energy levels of donor and acceptor components (to acquire the band gap of the photo active layer within 1.9–2.1 eV) for achieving comparatively elevated open-circuit voltage (*V*_OC_) in indoor, and good electrical charge transport capacity. As donor materials, a variety of organic polymers and small molecules (Fig. 3) were developed to satisfy all criteria. In addition, various fullerene (Fig. 4) and non-fullerene (Fig. 5) materials-based acceptors have also been developed. In terms of the active materials, the entire family of IOPVs can be classified into two types; fullerene-based IOPVs and non-fullerene-based IOPVs.

### 4.1. Fullerene acceptor-based IOPVs

Polymer donors and fullerene acceptors are used to develop active layers of first-generation OPVs. So far, there have been relatively few organic semiconducting materials like as poly(3-hexylthiophene-2,5-diyl) (P3HT), poly[[4,8-bis[(2-ethylhexyl)oxy]benzo[1,2-*b*:4,5-*b*′]dithiophene-2,6-diyl][3-fluoro-2-[(2-ethylhexyl)carbonyl]thieno[3,4-*b*]thiophenediyl]] (PTB7), poly[4,8-bis(5-(2-ethylhexyl)thiophen-2-yl)benzo[1,2-*b*;4,5-*b*′]dithiophene-2,6-diyl-*alt*-(4-(2-ethylhexyl)-3-fluorothieno[3,4-*b*]thiophene-)-2-carboxylate-2-6-diyl)] (PTB7-Th), poly[*N*-9′-heptadecanyl-2,7-carbazole-*alt*-5,5-(4′,7′-di-2-thienyl-2′,1′,3′-benzothiadiazole)], poly[[9-(1-octylnonyl)-9*H*-carbazole-2,7-diyl]-2,5-thiophenediyl-2,1,3-benzothiadiazole-4,7-diyl-2,5-thiophenediyl] (PCDTB-T), poly[4,8-bis(5-(2-ethylhexyl)thiophen-2-yl)benzo[1,2-*b*;4,5-*b*′]dithiophene-2,6-diyl-*alt*-(4-(2-ethylhexyl)-3-fluorothieno[3,4-*b*]thiophene-2-carboxylate-2-6-diyl)] (PBDTTT-EFT), and poly[(5,6-bis(2-hexyldecyloxy)benzo[*c*][1,2,5]thiadiazole-4,7-diyl)-*alt*-(5,50-(2,5-difluoro-1,4-phenylene)bis(thiophen-2-yl)] (PDTBTBz-2F_anti_) have been used as donors, whereas phenyl-C61-butyric acid methyl ester (PCBM) and indene-C60 bisadduct (ICBA) have been used as acceptors for these types of IOPVs. The performance parameters of various fullerene-based IOPVs (*i.e.*, binary) reported earlier are summarized in Table 1. In the early stages of IOPVs development, attaining higher *V*_OC_ posed a significant challenge. The *V*_OC_ value, measuring at a mere 0.43 V for IOPVs based on PC_60_BM, hindered their overall efficiency with a maximum PCE of only 9.59%. This limitation stemmed from the decreased energy level associated with the lowest unoccupied molecular orbit (LUMO) of the conventional PC_60_BM, as highlighted in Table 1. In response, a solution emerged by introducing a novel fullerene acceptor named ICBA. This breakthrough involved attaching two substitution groups onto the fullerene ball, effectively addressing the previously mentioned issue.^55,70,71^

**Table 1:** Summary of photovoltaic properties of previously reported fullerene-based indoor OPVs (binary)

Active layer	Light source	Luminance (lx)	*J*_SC_ [μA cm^−2^]	*V*_OC_ [V]	FF [%]	*P*_max_ [μW cm^−2^]	PCE [%]	Ref.
P3HT:PC_60_BM	FL	500	62.0	0.43	59.0	15.7	9.59	70
P3HT:PC_60_BM	LED	500	62.0	0.43	59.0	15.6	8.90	70
P3HT:ICBA	FL	500	50.0	0.73	62.0	22.5	13.7	70
P3HT:ICBA	LED	500	50.0	0.73	63.0	22.9	13.0	70
PBDTTT-EFT:PC_70_BM	FL	500	63.0	0.58	59.0	21.5	13.1	70
PBDTTT-EFT:PC_70_BM	LED	500	66.0	0.59	58.0	23.2	13.2	70
P3HT:PC_60_BM	FL	300	20.6	0.41	56.6	4.8	5.8	71
PCDTBT:PC_71_BM	FL	300	27.7	0.72	69.3	13.9	16.6	71
PTB7:PC_71_BM	FL	300	28.6	0.61	69.5	12.2	14.6	71
P3HT:ICBA	LED	500	40.4	0.67	68.3	—	10.8	55
P3HT:ICBA	LED	500	36.7	0.64	68.8	—	9.50	52
P3HT:ICBA	LED	1000	104	0.56	60.0	—	14.6	51
P3HT:ICBA	FL	500	45.5	0.65	59.0	—	9.3	51
P3HT:ICBA	Halogen	500	45.9	0.64	57.0	—	0.3	51
P3HT:PC_61_BM	FTube	500	35.9	0.47	62.4	—	7.48	108
P3HT:ICBA	LED	1000	68.2	0.71	64.9	36.4	13.0	48
P3HT:ICBA	LED	500	47.1	0.61	64.7	—	11.1	109
PTB7:PC_71_BM	LED	1000	87.6	0.56	69.3	—	12.3	75
PCDTBT:PC_71_BM	LED	300	28	0.70	46.0	9.0	8.7	93
PCDTBT:PC_71_BM	FL	300	53.5	0.74	53.0	20.7	11.5	93
PCDTBT:PC_71_BM	Incandescent	300	16.5	0.65	34.0	3.8	0.6	93
PCDTBT:PC_71_BM	FL	300	31.0	0.70	56.4	12.2	16.5	89
PCDTBT:PC7_1_BM	LED	300	31.0	0.70	56.6	12.5	16.2	89
P3HT:ICBA	LED	500	44.5	0.69	73.3	—	13.4	94
P3HT:ICBA	LED	500	28.4	0.68	63.3	12.4	7.3	44
P3HT:ICBA	LED	1000	63.0	0.72	61.6	28.0	10.0	44
PTB7-Th:PC_70_BM	LED	1000	133.4	0.66	65.8	57.8	13.9	74
P3HT:ICBA	LED	1000	85.5	0.71	66.8	—	14.1	95

It was then observed that ICBA-based IOPVs could exhibit higher *V*_OC_ (0.73 V) and PCE (13.7%) because of the high energy value associated with their LUMO level (Table 1). Other donor polymers, including such PTB7, have also been identified to have comparable PCE value. PTB7 has a greater absorption range (400–750 nm), although P3HT's absorption range is substantially smaller (350–650 nm). As a result, PTB7:PCBM-based devices may generate higher photocurrents. In comparison to P3HT:ICBA-based devices, PTB7:PCBM-based devices have a lower *V*_OC_ and fill factor (FF). However, these two types of devices are equivalent in terms of efficiency. Aside from PTB7, another polymer donor material known as PTB7-Th has shown to work well in IOPVs applications. Both PTB7 and PTB7-Th have an identical backbone conjugated structure. The chemical ligands attached to the benzodithiophene groups differ only in nature. In PTB7, it's an ether group, and in PTB7-Th, it's a thiophene group.^72^ In 2015, Mori and coworkers have used PTB7-Th:PCBM as an active layer of an IOPVs.^73^ The OPV was subjected to testing under an illumination of 186 lx from an LED source, revealing a noteworthy PCE of approximately 10.6%. Furthermore, an upper limit for the potential PCE was approximated at 21.3% for the same OPV (PTB7-Th:PC_70_BM) under the conditions of 186 lx LED illumination. Later, Marsal *et al.* have able to achieve ∼13.9% PCE (under 1000 lx LED lamp) value from a PTB7-Th:PCBM active layer based indoor OPV through the introduction an efficient ETL.^74^

Another instance of polymer:fullerene-based IOPV is based on a donor polymer named PCDTB-T.^71^ This polymer incorporates a carbazole donor unit, a benzothiadiazole acceptor unit, and two thiophene units as connecting elements. The inclusion of PCBM facilitates the generation of a notable *V*_OC_; in indoor conditions, this *V*_OC_ can achieve up to 0.7 V, a value that ranks among the highest observed for polymer:fullerene devices. Furthermore, devices based on PCDTB-T:PCBM exhibit an elevated shunt resistance (RP) of approximately kΩ cm^2^, providing an advantageous factor for achieving enhanced FF in environments with low light. In the course of this study, the researchers fabricated large-scale modules spanning an area of 100 cm^2^ and achieved a commendable PCE of 11.2%.

The innovation of new donor polymers for indoor applications improved the performance of indoor PCEs even further. Shim and colleagues, for example, created a donor polymer called PDTBTBz-2F_anti_,^75^ which has an absorption window that corresponds to indoor light. This polymer also has crystalline nano-fibrillar morphology with such a preferential face-on orientation for π–π stacking, allowing for improved electron injection and the construction of efficient thick film devices capable of enhancing light absorption and lowering leakage current. Because of these benefits, this polymer can achieve a high efficiency of 23.1% under a 1000 lx LED light. Surprisingly, under the 1 sun condition, the efficiency of OPV devices based on this polymer:fullerene combination is just 6.9%. This finding suggests that materials that aren't suited for outdoor OPV applications could be good for inside illumination.

A small molecule donor and a fullerene acceptor are used in another form of OPV. Unfortunately, there have been limited studies on the application of small-molecule donor and fullerene acceptor-based OPVs for low-intensity indoor light harvesting. Tsoi *et al.* revealed in 2018 that they harvested energy from a 1000 lx FL lamp using a benzodithiophene terthiophene rhodamine (BTR) small molecule as a donor and a PC_71_BM acceptor-based OPV.^76^ They chose this material as the donor because of its interesting morphology (arising from the special arrangement of the alkyl chain) and sufficiently higher optical energy band gap (1.80 eV) (suitable for indoor light harvesting). They were able to achieve a balance between crystallization and phase separation by optimizing the active layer's solvent vapour annealing duration, and they were able to produce a very high PCE (>28%) while the device was operated under 1000 lx FL lamp. Additionally, the device produced a very high maximum power density value (78.2 μW cm^−2^). Continued research into these sorts of donor and acceptor material combinations is likely to result in IOPVs with greater PCE and longer lifespans.

### 4.2. Non-fullerene acceptor-based IOPVs

In the realm of OPVs, a recent and remarkable breakthrough has emerged – the advent of a novel class of acceptor materials known as non-fullerene acceptors. Unlike traditional fullerene acceptors, which suffer from limited absorption in the visible spectrum, non-fullerene acceptors offer a solution to this challenge. Moreover, their unique feature lies in the ability to finely adjust their electronic structure, enabling the attainment of desired energy band gaps tailored to different artificial light sources. This flexibility facilitates the modulation of *V*_OC_, a crucial parameter in OPV performance. Importantly, non-fullerene acceptors exhibit the potential to exhibit high light absorption within the visible range, coupled with the capacity to fine-tune their frontier molecular orbital levels. This characteristic proves invaluable in enhancing *V*_OC_, particularly under the constraints of low-intensity indoor lighting conditions, by minimizing energy losses. As a result of these exceptional attributes, non-fullerene acceptors have emerged as highly promising contenders for the advancement of IOPVs. During the last few years, several well-behaved non-fullerene acceptors such as 3,9-bis[5,6-dichloro-1*H*-indene-1,3(2*H*)dione]-5,5,11,11-tetrakis(4-hexylphenyl)-dithieno[2,3-*d*:2′,3′-*d*′]-*s*-indaceno[1,2-*b*:5,6-*b*′]dithiophene (IO-4Cl), 3,9-bis(2-methylene-((3-(1,1-dicyanomethylene)-6,7-difluoro)-indanone))-5,5,11,11-tetrakis(4-hexylphenyl)-dithieno[2,3-*d*:2′,3′-*d*′]-*s*-indaceno[1,2-*b*:5,6-*b*′]dithiophene (IT-4F), 3,9-bis(4-(1,1-dicyanomethylene)-3-methylene-2-oxo-cyclopenta[*b*]thiophen)-5,5,11,11-tetrakis(4-hexylphenyl)-dithieno[2,3-*d*′:2,3-*d*′]-*s*-indaceno[1,2-*b*:5,6-*b*′]-dithiophene (ITCC), 3,9-bis(2-methylene-((3-(1,1-dicyanomethylene)-6/7-methyl)-indanone))-5,5,11,11-tetrakis(4-hexylphenyl)-dithieno[2,3-*d*:2′,3′-*d*′]-*s*-indaceno[1,2-*b*:5,6-*b*′]dithiophene (ITIC-M), and poly[5,10-bis(2-decyltetradecyl)-2-(5-(2,5-difluoro-4-(5-methylthiophen-2-yl)phenyl)thiophen-2-yl)-4,4,9,9-tetrafluoro-7-methyl-4,5,9,10-tetrahydro-3*a*,5,8*a*,10-tetraaza-4,9-diborapyrene-3*a*,8*a*-diium-5,11-diuide] (PBN-10) have been developed and used in OPVs for indoor applications.^36,43,77–84^ In this subsection, we shall discuss the recent studies on the application of various non-fullerene acceptor-based IOPVs. The performance parameters of various non-fullerene-based IOPVs (binary) reported earlier are summarized in Table 2.

**Table 2:** Summary of photovoltaic properties of previously reported non-fullerene-based indoor OPVs (binary)

Active layer	Light source	Luminance (lx)	*J*_SC_ [μA cm^−2^]	*V*_OC_ [V]	FF [%]	*P*_max_ [μW cm^−2^]	PCE [%]	Ref.
CD1:PBN-10	LED	1000	105.0	1.14	65.4	78.0	21.7	77
CD1:ITIC	LED	1000	107.0	0.77	67.5	—	15.4	77
PBDB-T:IO-4Cl	LED	200	18.2	1.03	71.5	13.4	22.2	80
PBDB-T:IO-4Cl	LED	500	45.1	1.07	76.8	37.1	24.6	80
PBDB-T:IO-4Cl	LED	1000	90.6	1.10	79.1	78.1	26.1	80
PBDB-TS:IT-4F	FL	1000	125.5	0.48	36.2	22.1	7.0	78
PBDB-TS-3Cl:IT-4F	FL	1000	123.8	0.66	72.8	60.2	19.4	78
PBDB-TS-4Cl:IT-4F	FL	1000	129.3	0.66	74.3	64.0	20.7	78
PBDB-TF:IT-M	LED	500	53.4	0.88	69.5	75.4	22.8	79
PM6:Y6-O	LED	1650	245	0.84	76.0	—	30.8	36
P3TEA:FTTB-PDI4	LED	1650	196.0	1.03	67.0	—	26.6	36
PM6:IT-4F	LED	1000	113.0	0.71	78.0	62.8	20.8	81
PM6:ITCC	LED	1000	95.8	0.96	72.0	66.5	22.0	81
PBDB-TSCl:IT-4F	FC	500	117.9	0.66	77.0	29.05	21.5	41
TPD-3F:IT-4F	FC	1000	361.0	3.21	71.0	40.2	21.8	82
PM6:Y-Th2:Y6	LED	1000	320.1	0.70	74.0	163.0	22.7	43
D18:FCC-Cl	LED	500	61.6	0.94	79.5	45.8	28.8	85

Because of its broad (1.80 eV) optical energy band gap, IO-4Cl is one of the best non-fullerene acceptors for IOPVs to harvest light energy generated by LEDs. In 2019, Hou and coworkers developed this fullerene-free semiconductor, which was designed as an acceptor–donor–acceptor structure.^80^ They combined it with a polymer donor known as poly[(2,6-(4,8-bis(5-(2-ethylhexyl-3-fluoro)thiophen-2-yl)-benzo[1,2-*b*:4,5-*b*′]dithiophene))-*alt*-(5,5-(1′,3′-di-2-thienyl-5′,7′-bis(2-ethylhexyl)benzo[1′,2′-*c*:4′,5′-*c*′]dithiophene-4,8-dione))] (PBDB-TF) to create a photo-active layer for indoor OPVs. Under the illumination of a 1000 lx (2700 K) LED, an IOPV with a 1 cm^2^ active layer exhibited remarkable attributes, boasting an elevated PCE of 26.1% and a notably higher *V*_OC_ of 1.10 V. Impressively, this OPV showcased exceptional stability, maintaining consistent photovoltaic performance throughout 1000 hours of uninterrupted LED exposure. Within the core of IO-4Cl, the electron-donating fragment molecule ITIC assumes a pivotal role. Constructed around a stiff core comprising two sp^3^-hybridized carbon atoms, this molecule's propensity for self-aggregation is mitigated by the steric hindrance introduced by aromatic side chains. The incorporation of fused rings further curbs rotational perturbations. Consequently, this configuration substantially reduces reorganization energy and mitigates voltage loss when the organic photovoltaic device operates under the conditions of low-intensity indoor lighting.

Another noteworthy non-fullerene acceptor material goes by the name of IT-4F. This material, like ITIC, is founded upon the same foundation, with the addition of four fluorine atoms at its terminal group. These fluorine atoms introduce an electron-attracting influence, which contributes to heightened crystallinity and enhanced π–π stacking within the active layer. Furthermore, they play a role in shifting the acceptor's LUMO and HOMO levels downwards, thereby elevating the FF of the device. When employed as an acceptor in OPVs, IT-4F exhibited impressive performance, achieving a PCE exceeding 14% under standard 1 sun illumination. However, owing to a reduction in voltage, OPVs utilizing the IT-4F acceptor demonstrated inferior performance in scenarios of low-intensity indoor lighting. For example, Hou and colleagues used PBDB-TF:IT-4F as the active layer to build very efficient IOPVs, achieving a maximum PCE of 20.8% for a 1000 lx 2700 K LED illumination.^81^ Although achieving a PCE surpassing 20% and showcasing consistent performance throughout prolonged operation under conditions of low-intensity indoor lighting, the inability to achieve an even higher PCE value can be attributed to the lower *V*_OC_ of 0.71 V. This lower *V*_OC_ could potentially be traced back to the comparatively reduced optical energy bandgap associated with IT-4F.

Son *et al.* tried to improve the PCE value of IT-4F acceptor-based OPVs by introducing a new donor material called 2-ethylhexyl thiophene-substituted benzodithiophene, 1,3-bis(2-ethylhexyl)-5,7-di-(thiophene-2-yl)benzo[1,2-*c*:4,5-*c*′]dithiophene-4,8-dione (PBDB-TSCl).^41^ When compared to PBDB-TF, the fluorine substitution in this material is replaced with chlorine. A sulfur atom is also sandwiched between the 2-ethylhexyl alkyl chain and the thiophene unit. As a result, the acceptor outperformed PBDB-TF in charge transport ability. Under a 500 lx FL light, the OPV based on PBDBTSCl:IT-4F achieved a maximum PCE of 21.5%. Despite the device's very reliable performance, the PCE was not significantly enhanced due to the reduced *V*_OC_ (0.63 V).

Recently, Yin *et al.* reported a record high PCE (>30%) from a non-fullerene based IOPVs.^36^ The device was built around a PBDB-TF donor and a Y6O acceptor, both of which are based on 2,2′-((2*Z*,2′*Z*)-((12,13-bis(2-ethylhexyl)-3,9-diundecyl-12,13-dihydro-[1,2,5]thiadiazolo[3,4*e*]thieno[2′′,3′′:4′,5′]thieno[2′,3′:4,5]pyrrolo[3,2-g]thieno[2′,3′:4,5]thieno[3,2-*b*]indole-2,10-diyl)bis(methanylylidene))bis(5,6-difluoro-3-oxo-2,3-dihydro-1*H*-indene-2,1-diylidene))dimalononitrile (Y6) by substituting an alkyl chain at the β-position of the merged thiophene unit with an alkoxy side chain. In comparison to the indoor illumination spectrum, the absorption range of Y6 is often up to 940 nm, which is excessively red-shifted. As a result, the alkoxy substitution aided to blue-shift the acceptor's absorption by increasing its HOMO and LUMO levels. The device has an extremely high *V*_OC_ (0.84 V) and FF (0.76%), as well as a record-breaking PCE value. The same team has designed FCC-Cl, a new high-performance non-fullerene acceptor with a desirable optical band gap for IOPVs, by coupling a weak electron-donating core with a moderate electron-withdrawing end group. Because of the high crystallinity, wide energy band gap and high absorption coefficient of FCC-Cl, the FCC-Cl acceptor-based OPVs demonstrated a very high PCE (28.8%) under the light of 500 lux LED (2600 K).^85^ Thick-film devices based on FCC-Cl were also created, and devices with a thickness of 300 nm may still achieve a PCE of 26.5% under LED at 1000 lx. IOPV devices' high thickness tolerance is a desired attribute for roll-to-roll large-area printing processes.

A unique donor–acceptor combination (polymer donor and polymer non-fullerene acceptor) was been employed as an active layer for IOPVs. Liu *et al.* reached 27.4% PCE (under the irradiation of a FL lamp) and exceptionally high *V*_OC_ (1.16 V) in 2019 from a poly[4-(5-(4,8-bis(5-((2-butyloctyl)thio)thiophen-2-yl)-6-methylbenzo[1,2-*b*:4,5-*b*0]dithiophen-2-yl)thiophen-2-yl)-5,6-diuoro-2-(2-hexyldecyl)-7-(5-methylthiophen-2-yl)-2*H*-benzo[*d*][1,2,3]triazole] (CD1) donor and poly [5,10-bis(2-decyltetradecyl)-2-(5-(2,5-difluoro-4-(5-methylthiophen2-yl)phenyl)thiophen-2-yl)-4,4,9,9-tetrafluoro-7-methyl-4,5,9,10-tetrahydro-3*a*,5,8*a*,10-tetraaza-4,9-diborapyrene-3*a*,8*a*-diium-5,11-diuide] (PBN-10) acceptor-based IOPVs.^77^ Considering the donor's extremely high HOMO level, the device produced extremely high *V*_OC_. Furthermore, because of the polymer nature of the donor and acceptor substances, the device displayed greater mechanical flexibility and stretchability.

In 2022, Yan *et al.* have reported a series of large-bandgap (>1.70 eV) NFAs named FCC-Cl-C8, FCC-Cl-4Ph and FCC-Cl-6Ph by modifying the alkyl side chains with alkylphenyl chains partially or completely.^86^ In their work it has been observed that the bulky alkylphenyl side chains can finely tune the absorption properties of the NFAs and also affect their morphological properties. Interestingly it has been observed that the best-performing NFA is the one (named FCC-Cl-4Ph) with partial alkyl and alkylphenyl substitutions, which blue-shift the absorption of the NFAs while minimizing the negative morphological effect of the bulky alkylphenyl chains. As a result, FCC-Cl-4Ph can achieve excellent indoor efficiencies over 29% under a 3000 K LED lamp at 1000 lx and show better solution processability over FCC-Cl-C8. More importantly, FCC-Cl-4Ph can maintain high indoor performance (29.7–26.8% at 1000 lx) under a wide range of indoor lighting spectra (2600, 3000, 4000, and 6500 K LED lamps), which should be due to the blue-shifted spectra of FCC-Cl-4Ph and better matching with various indoor conditions. This work reveals an interesting structure–property relationship and offers useful strategies for the further design of NFAs toward efficient IOPV devices.

Hou and coworkers, recently a designed and synthesized a series of polymers (PB3, PB4 and PB5) based on thiadiazole (TDZ), 4,8-bis(5-(2-ethylhexyl)thiophen-2-yl)benzo[1,2-*b*:4,5-*b*′]dithiophene (BDT-T) and fluorinated BDT-T (BDT-T-2F) units based on a random polymerization strategy.^37^ The terpolymers have demonstrated wide optical bandgaps larger than 2.0 eV. Compared to the host polymer PB2, the terpolymers have exhibited downshifted energy levels and enhanced crystallinities, which show much lower energetic disorders. After blending with a wide bandgap non-fullerene acceptor FTCC-Br, the optimized terpolymer have shown a much faster charge transfer rate than PB2. Importantly, the OPV cell based on PB3:FTCC-Br has achieved a high PCE of over 15% with an *V*_OC_ of 1.06 V under 1 sun condition. The PB4:FTCC-Br-based cell has demonstrated a PCE of 14.79% with a high *V*_OC_ of 1.09 V. Furthermore, the PB3:BTP-eC9-based cell has exhibited a PCE of 18.28%. Besides, the PB4:FTCC-Br-based cell with an effective area of 1.0 cm^2^ has exhibited a PCE of over 31% under 2700 K illumination of 1000 lx. The results indicate that the ternary copolymerization strategy is an effective way to fine-tune the opto-electronic properties of highly efficient polymer donors for versatile photovoltaic applications. Later the same group have achieved over 33% PCE value from PB2:FCC-Cl active material based IOPV under concentrated indoor light.^87^ Additionally, the device has exhibited superior stability (extrapolated *T*_80_ over 30 000 h) under indoor light.

The correlation between molecular structure and photovoltaic performance is lagging for constructing high-performance IOPV cells. Recently, Hou *et al.* investigated this relationship in depth by employing two medium-bandgap NFAs. The newly synthesized NFA of FTCC-Br exhibited a similar bandgap and molecular energy level, but a much stronger dipole moment and larger average electrostatic potential (ESP) compared with ITCC. After blending with the polymer donor PB2, the PB2:ITCC and PB2:FTCC-Br blends exhibit favorable bulk-heterojunction morphologies and the same driving force, but the PB2:FTCC-Br blend exhibited a large ESP difference. In OPV cells, the PB2:ITCC-based device produces a PCE of 11.0%, whereas the PB2:FTCC-Br-based device gives an excellent PCE of 14.8% with an *V*_OC_ of 1.05 V, which was the highest value among OPV cells with *V*_OC_ values above 1.0 V. When both acceptor-based devices worked under a 1000 lx of 3000 K light-emitting diode, the PB2:ITCC-based 1 cm^2^ device yields a good PCE of 25.4%; in contrast, the PB2:FTCC-Br-based 1 cm^2^ device outputted a record PCE of 30.2%. These results suggested that a large ESP offset in photovoltaic materials was important for achieving high-performance OPV cells.^88^

## 5. Various approaches for improving performance of IOPVs

### 5.1. Multi donor–acceptor-based IOPVs

Traditional binary donor–acceptor-based active layers often face challenges due to inadequate crystallinity, suboptimal morphology, and reduced absorption coefficients. These limitations hinder the attainment of elevated *J*_SC_, and consequently, hinder the overall PCE achievable in indoor applications. To address this concern, efforts have been directed towards enhancing the active materials' characteristics in IOPVs through the incorporation of multiple donors and acceptors. This innovative approach offers certain advantages, including the potential for thinner device profiles, circumvention of current–voltage matching constraints, and more. Nonetheless, achieving optimized absorption and charge-injection capabilities necessitates precise tuning of their bulk heterojunction (BHJ) morphology. Moreover, parameters such as electrical energy levels, bandgap, solubility, and compatibility between various materials must be thoughtfully considered during the device fabrication process. Table 3 summarizes the performance characteristics of several multiple donor–acceptor-based IOPVs previously described.

**Table 3:** Summary of photovoltaic properties of previously reported multiple donor–acceptor-based OPVs

Active layer	Light source	Luminance (lx)	*J*_SC_ [μA cm^−2^]	*V*_OC_ [V]	FF [%]	*P*_max_ [μW cm^−2^]	PCE [%]	Ref.
PBDB-T:PTB7-Th:ITIC-Th:PC7_0_BM	LED	500	43.7	0.63	64.5	—	10.5	90
PBDB-T:PTB7-Th:ITIC-Th:PC7_0_BM	LED	1000	99.2	0.67	64.8	—	15.4	90
PBDB-T:PTB7-Th:ITIC-Th:PC7_0_BM	FL	500	49.5	0.64	65.5	—	10.8	90
PBDB-T:PTB7-Th:ITIC-Th:PC7_0_BM	LED	1000	114.0	0.67	63.0	—	14.6	90
PCDTBT:PTB7:PC_61_BM:PC_71_BM	LED	500	44.0	0.58	71.0	18.0	10.8	65
PTB7:PC_61_BM:EP-PDI	LED	500	57.1	0.65	68.5	—	15.6	110
PCDTBT:PDTSTPD:PC_71_BM	FL	300	33	0.73	63.5	20.8	15.4	89
PCDTBT:PDTSTPD:PC_71_BM	LED	300	33	0.73	61.0	19.0	14.6	89

**Fig. 6:**
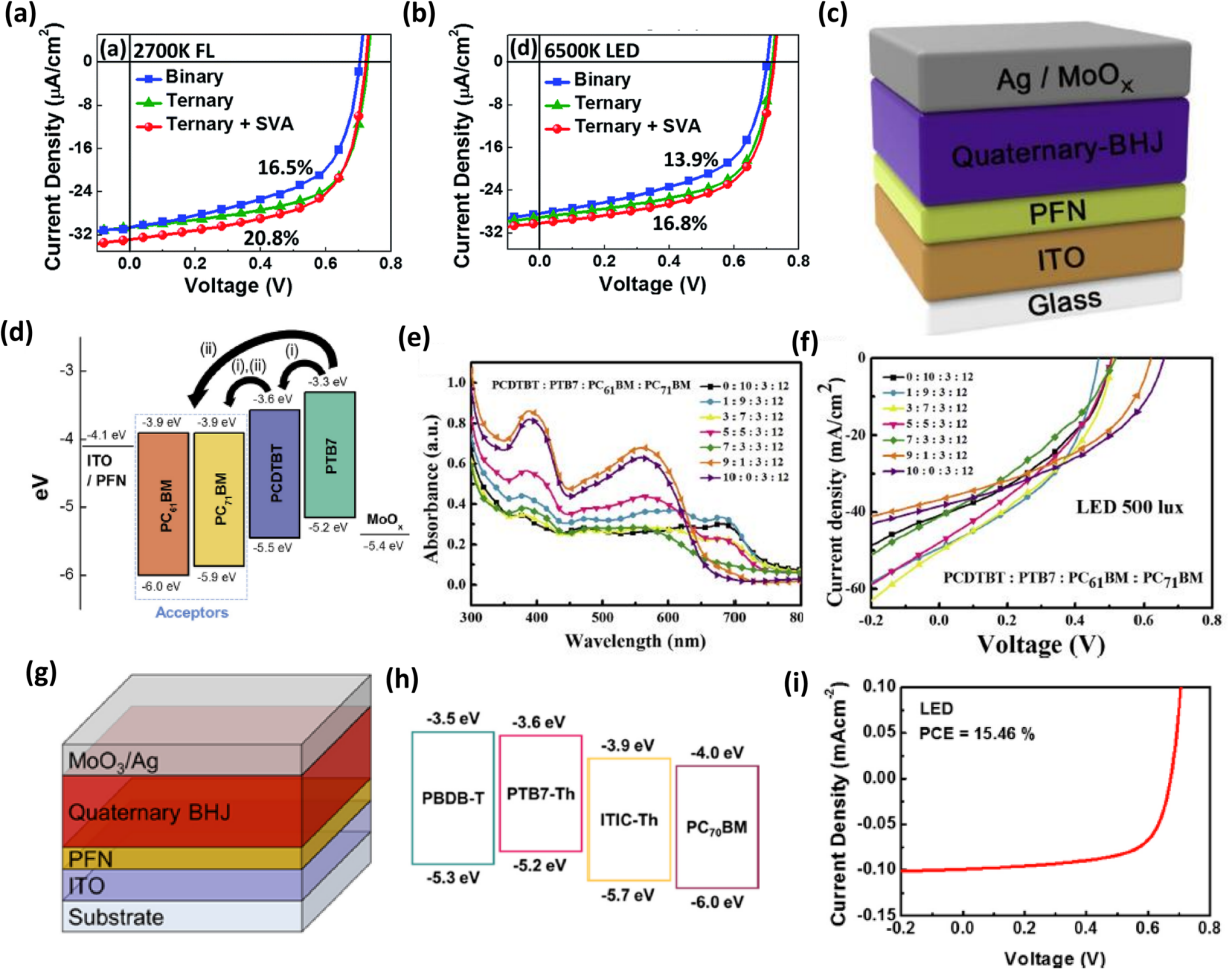
*J*–*V* characteristics of binary PCDTBT:PC_71_BM and ternary PCDTBT:PDTSTPD:PC_71_BM OPVs under (a) 300 lx FL lamp (2700 K) and (b) 300 lx LED illumination (6500 K); [reproduced with permission.^89^ Copyright 2018, The Royal Society of Chemistry]. (c) Quaternary OPV device construction, (d) organic compound energy band levels in the BHJ and other layers, (e) BHJ absorbance with varied composition weight ratios, (f) experimentally determined *J*–*V* curves of quaternary OPVs with various composition weight ratios under 500 lx white LED illumination; [reproduced with permission.^65^ Copyright 2019, Elsevier]. (g) ST Q-OPV schematic diagram and (h) energy level, based on two polymer donors, one fullerene acceptor, and one non-fullerene acceptor, (i) *J*–*V* curve of the ST Q-OPV under 1000 lx LED illumination; [reproduced with permission.^90^ Copyright 2019, Elsevier].

So *et al.* created a highly efficient ternary IOPV in 2018 using PCDTBT and PDTSTPD as donor substances and PC_71_BM as the acceptor material.^89^ Under the irradiation of a 2700 K, 300 lx FL lamp, the ternary OPV had a maximum PCE of 19.8% (Fig. 6a). The ternary OPV had a very high *J*_SC_ value, which was mostly due to the active layer's absorption window being expanded up to the near infrared range. To enhance device performance even more, the active layer was annealed for 30 s after spin coating. Under the same illumination conditions, the device (solvent vapour annealing-treated) exhibited 20.8% PCE value along with a *V*_OC_, FF, and *J*_SC_ of 0.73 V, 63.5%, and 33.3 mA cm^−2^ respectively (Fig. 6a). Surprisingly, when illuminated by other light sources with greater color temperatures, the same device worked very inadequately (Fig. 6b), leading to incompatibilities between the light source's irradiance spectra and the active layer's absorption spectra.

Recently, Shim *et al.* have reported the utilization of a quaternary bulk heterojunction to harvest indoor light energy. Because the HOMO and LUMO values of the component materials were well matched, they selected PCDTBT and PTB7 as donors and PC_61_BM and PC_71_BM as acceptor materials for the active layer (Fig. 6c) (Fig. 6d). They showed that the performance of a quaternary IOPVs is largely reliant on the blend ratio of the component materials of active layers, and that with an optimal blend ratio, a maximum PCE value from a quaternary IOPVs can be reached (Fig. 6e and f). Under the illumination of a 500 lx white LED with a composition ratio of 5 : 5 : 3 : 12, they achieved a maximum PCE value of 10.6% (PCDTBT : PTB7 : PC_61_BM : PC_71_BM). At this ideal composition ratio, the device exhibited very high shunt resistance and low series resistance, resulting in considerable charge extraction under low-intensity light conditions.^65^

Besides opaque IOPVs, highly efficient quaternary active material-based semi-transparent indoor IOPVs have also been reported. In 2019, Ko and coworkers developed a semi-transparent IOPVs based on quaternary blends (PBDB-T:PTB7-Th:ITIC-Th:PC_70_BM) (Fig. 6g and h). They proved the device's practical applicability in four-terminal (4T) tandem OPVs.^90^ The semitransparent quaternary OPV demonstrated a very high PCE value (15.46% when illuminated by a 1000 lx LED light) (through the minimization of energy loss and maximization of absorption coefficient) while keeping excellent transparency and the ability to incorporate multiple colors (Fig. 6i).

Recently, Ko and coworkers discussed the sequential deposition method, as an alternative strategy for the facile realization of high-performance multicomponent BHJ OPVs for indoor applications. The sequential deposition improved the collection of indoor light, modified the molecular alignment of polymers, and reduced the hole trap-state density and charge recombination loss in the quaternary BHJ films. The sequentially processed quaternary OPVs exhibited superior PCEs approaching 26.06% under diverse indoor irradiation environments, while neither compositional optimization nor additional treatment steps were implemented. The sequential deposition method can be a platform to simplify the fabrication process of multicomponent BHJ OPVs while exploiting the advantages offered by the multiple materials and their synergistic benefits to comply with the requirements of efficient indoor applications. Further investigation of the molecular evolution during sequential deposition can enrich this methodology and is currently underway.^91^

The review study discerns that despite the considerable promise of this strategy in advancing highly efficient IOPVs, only a scant number of endeavors have been undertaken thus far.

### 5.2. Utilization of interlayer (HTL, ETL)

**Fig. 7:**
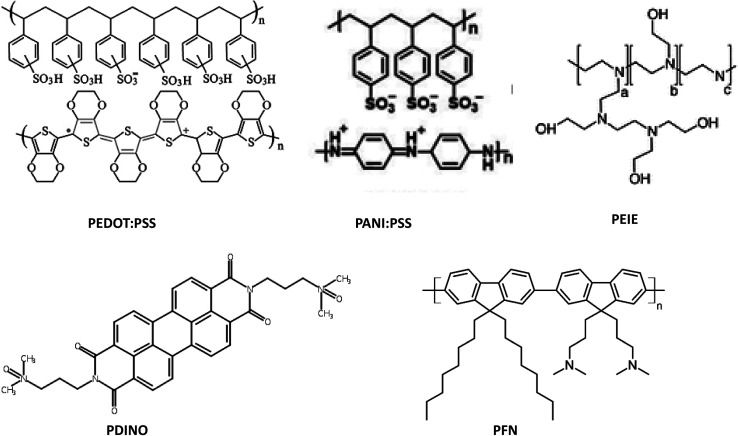
Chemical structures of several materials used as indoor OPV interlayers (HTL or ETL).

In the realm of OPVs, two distinctive types of interlayers come into play. The first is the HTL, strategically positioned between the device's anode and the active layer. The second is the ETL, situated between the device's cathode and the active layer. The integration of these interlayers serves multifaceted purposes, encompassing the mitigation of defect states and potential surface charge recombination, as well as the reduction of energy barriers existing between the electrode and the OPV's active layer. As the landscape stands, diverse semiconducting materials, spanning both organic and inorganic realms, have been harnessed to fulfill HTL and ETL roles in various OPV configurations. The chemical structures of various materials used as ETL and HTL of various IOPVs are presented in Fig. 7. In this subsection, we will discuss the development of various HTLs and ETLs for the improvement of various IOPVs.

In 2013, Hegger *et al.* tried to optimize the performance of a DTS(FBTTh_2_)_2_:PC_71_BM active layer-based OPV operating under various light sources having variable intensities through the regulation of the thickness of its ETL formed by calcium (Ca).^92^ They observed that first-order charge recombination regulated the *J*–*V* characteristic curves between the short-circuit and maximum power point (MPP) at an appropriate ETL thickness, and that this control was then translated to biomolecular charge recombination in between MPP and open-circuit circumstances. Because of the lack of Shockley–Read–Hall recombination and strong charge-collection efficiency, the device had a very high FF value (73%).

**Fig. 8:**
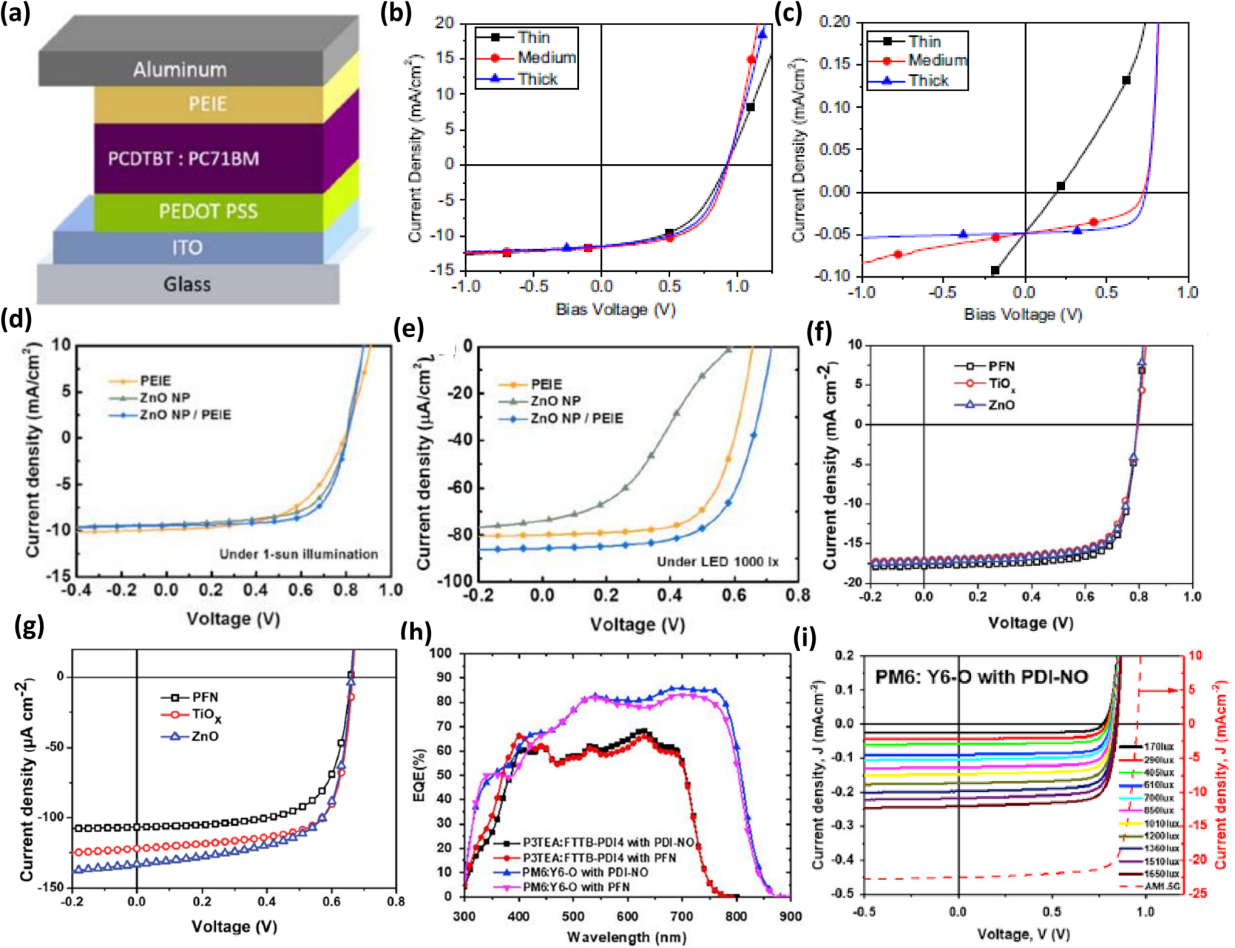
(a) An indoor OPV architecture based on PEIE ETL and PCDTBT:PC_71_BM active layer, *J*–*V* characteristics of three OPVs with varying PEIE thicknesses operating under (b) 1 sun and (c) low-intensity indoor light; [reproduced with permission.^93^ Copyright 2016, Elsevier]. *J*–*V* characteristics of OPVs with PEIE, ZnO NP, and ZnO NP/PEIE ETL under (d) 1 sun condition and (e) 1000 lx LED light; [reproduced with permission.^95^ Copyright 2019, Elsevier]. *J*–*V* characteristics of OPVs with PFN, TiO_*x*_, and ZnO ETL operating under (f) 1 sun and (g) 1000 lx LED illumination; [reproduced with permission.^74^ Copyright 2020, The Royal Society of Chemistry]. (h) EQE spectra of OPVs with PM6:Y6-O and P3TEA:FTTB-PDI4 active layers and PDI-NO and PFN ETLs, (i) representative *J*–*V* curves of an OPV with PM6:Y6-O active layer and PDI-NO ETL operating under different light sources; [reproduced with permission.^36^ Copyright 2020, Cell Press].

In 2016, Lechêne *et al.* studied the utilization of ethoxylated polyethylenimine (PIEE) as an ETL for IOPVs (PCDTBT:PC_70_BM active-layer-based) (Fig. 8a).^93^ They demonstrated that maximum PCE from an IOPVs can be achieved through device optimization for a particular light source. More specifically, minimization of dark current of an IOPVs is essential to achieve enhanced PCE from an indoor OPV. The PCE of an OPV optimized in the 1 sun condition was 6.2%, while the PCE of the very same device under low-intensity indoor light was 5.2% (Fig. 8b). Interestingly, when the OPV was tuned for indoor light, it showed 7.6% PCE under artificial indoor light illumination (Fig. 8c).

Shim and colleagues then investigated the relationship between the ETL's electrical characteristics and the parasitic resistance of an IOPV (PEIE ETL and P3HT:ICBA active-layer-based).^94^ PEIE is an insulator but it has an amine group, which can donate electrons on the surface of the electrode and form dipoles. The ensuing dipole moment lowers the work function on the conductor surface, causing a vacuum level downshift. The thickness of the ETL was adjusted by varying its thickness from 1.7 to 22.8 nm. Under the 1 sun condition, PCE (maximum) value of 4.7 ± 0.3% was acquired for a 2.2 nm thick PEIE layer. Additionally, when the thickness of the PEIE film rose, the PCE and FF of the OPV steadily decreased due to the rise in *R*_S_. Under the irradiation of a 500 lx LED light, however, the OPVs with a thicker PEIE layer (8.5 nm) efficiently harvested photon energy (PCE ≈ 13% and FF > 73%). The study concluded that a distinct cell design method is required to maximize OPV performance under low-intensity indoor lighting.

In 2019, the same group reported the application of an ultra-thin ZnO layer (modified by PEIE) as a highly efficient ETL of an inverted structured OPV (P3HT:ICBA active layer based).^95^ Under the irradiance of a 1000 lx LED lamp, an OPV with only a ZnO nanoparticle-based ETL could have a very poor PCE (5.6%) due to the formation of a Schottky barrier caused by energy level inconsistency between the insufficiently low work function of the ZnO nanoparticles and the electron affinity of the ICBA. Under the same illumination circumstances, an OPV with a PIEE-modified ZnO layer as the ETL had a very high PCE value (14.1%). It was suggested that the PEIE adjustment created a dipole moment, which led in an adequate work function decrease and a remarkable improvement in the IOPVs performance (Fig. 8d and e).

Very recently, Ylikunnari and coworkers tested the potential of ZnO and SnO_2_ as the ETLs in flexible OPVs (PV2001:PC_61_BM active-layer-based), for operating under low-intensity indoor light.^96^ They observed that ZnO ETL-based OPVs could exhibit a higher PCE than a SnO_2_ ETL-based OPV under the 1 sun condition. However, when exposed to low-intensity indoor light, the SnO_2_ ETL-based OPVs (PCE > 13% at 800 lx (*i.e.*, a FL lamp)) performed better than the ZnO ETL-based OPVs. They concluded that the performance level of ETLs under various illuminating conditions is correlated to the adjustment of barrier height (related to charge transport) due to the change in illumination conditions (especially the presence or absence of UV light). ZnO is known to be UV-light sensitive; therefore, under the 1 sun condition, the UV light reduced the energy barrier (related to electron transport) and improved the electron extraction ability of the ZnO layer. Consequently, the ZnO ETL-based OPV exhibited better performance under the 1 sun condition. By contrast, the low-intensity indoor light is UV-region free; therefore, during the indoor operation of ZnO ETL-based OPV, the effect of UV light irradiation was missing, so the device exhibited poor performance. Interestingly, for SnO_2_, this type of effect (due to UV irradiation) was missing. As a consequence, under ambient light conditions, SnO_2_ ETL acquired more electrons, making a much more potential candidate as ETLs for indoor applications over ZnO.

According to Marsal and colleagues, the performance of an inverted structured OPV is greatly influenced by lighting conditions.^74^ For this purpose, they fabricated an inverted structured OPV (PTB7-Th:PC_70_BM active-layer-based) by employing three different types of solution-processed ETLs (PFN, TiO_*x*_, and ZnO). It was found that the OPV with poly [(9,9-bis(3′-(*N*,*N*-dimethylamino)propyl)-2,7-fluorene)-*alt*-2,7-(9,9-dioctylfluorene)] (PFN) as the ETL had the greatest PCE (10.55%) value under 1 sun condition and the lowest performance under low-intensity LED light (250–2000 lx 2700 K) illumination. The ZnO ETL-based OPV had a lower PCE (10.03%) under the 1 sun condition, but it had improved indoor efficiency with such a PCE of 13.94% under a 1000 lx LED lamp and 16.49% under a 1750 lx LED lamp (Fig. 8f and g). The result was further demonstrated on the basis of the variation of parasitic resistance of various ETL-based OPVs.

Yin *et al.* have explored the importance of energy band alignment between the ETL and acceptors in the operation of an IOPVs.^36^ They employed PM6:Y6-O as an active layer and 2,9-bis[3-(dimethyloxidoamino)propyl]anthra[2,1,9-*def*:6,5,10-*d*′*e*′*f*′]diisoquinoline-1,3,8,10(2*H*,9*H*)-tetrone (PDINO) or PFN as the device's ETL. The PFN and PDINO ETL-based OPVs demonstrated similar PCE values (16.1% (PFN) and 16.4% (PDI-NO)) under 1 sun condition, however the devices behaved differently under low-intensity indoor lighting. The PDINO ETL-based OPV demonstrated extremely high PCE (30.8%) when illuminated with a 1650 lx LED, but the PFN ETL-based OPV had lower PCE (22%) (Fig. 8h and i). They suggested that the difference was due to the variation in band alignments in the two devices. The energy associated with the HOMO level of PDINO and PFN is −6.21 eV and −5.61 eV respectively. As a consequence, PDINO could prevent the movement of holes more efficiently than the PFN layer, resulting in decreased leakage current for low-intensity light illumination.

Recently, Kim *et al.* fabricated ZnO and gallium (Ga)-doped ZnO (GZO) ETL-based inverted structured OPVs with PBDB-T:PC_70_BM active layer and evaluated them under LED (1000 lx).^97^ To optimize the doping concentration, they varied the concentration of Ga in the GZO. The findings demonstrated that only by doping the ETL of the inverted structured OPV, the PCE may be significantly enhanced. Under a 1000 lx LED illumination, the device using undoped ZnO as the ETL showed a maximum of 15.56% PCE. The OPV with Ga-doped ZnO as the ETL with an optimized concentration of Ga (2 at%) exhibited a maximum of 23.42% PCE under a 1000 lx LED.

**Fig. 9:**
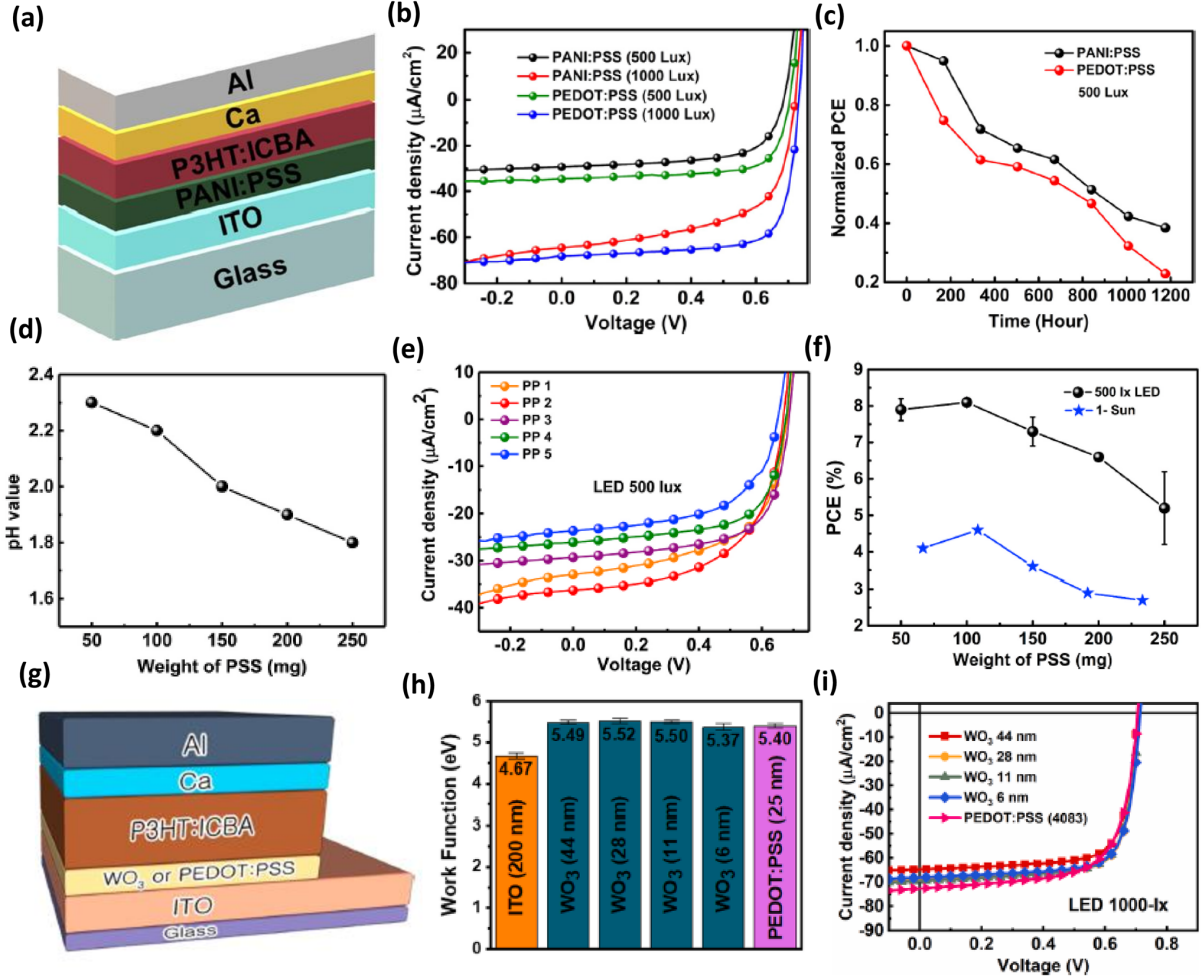
(a) Architecture of an indoor OPV based on PANI:PSS HTL and P3HT:ICBA active layer, (b) *J*–*V* characteristics of the OPV operating under 500 lx and 1000 lx LED, (c) variation of normalized PCE of OPVs (PEDOT:PSS and PANI:PSS HTL-based) with their lifespan; [reproduced with permission.^44^ Copyright 2020, Elsevier]. (d) Variation of pH value of PANI:PSS as per its concentration of PSS, (e) representative *J*–*V* characteristics of an OPV operating under the illumination of 500 lx LED, (f) variation of PCE value of OPVs (operating under 500 lx LED and 1 sun condition) with the doping concentration of its HTL; [reproduced with permission.^46^ Copyright 2020, Elsevier]. (g) Architecture of an indoor OPV with WO_3_ HTL and P3HT:ICBA active layer, (h) comparison of work function value of ITO, WO_3_ and PEDOT:PSS, (i) representative *J*–*V* characteristics of OPVs (having different HTLs) operating under the illumination of 1000 lx LED; [reproduced with permission.^48^ Copyright 2020, Elsevier].

Although a significant number of inorganic and organic semiconductors have been tested as ETLs of IOPVs, very few HTLs have been tested.^98–101^ Generally, PEDOT:PSS is used as the HTL of IOPVs because of its easy processability, water stability, superior hole transport ability, and desired work function (approximately 5.2 eV). However, it has a number of significant disadvantages, including high acidity, UV radiation sensitivity, and a high price. The device's lifespan is significantly shortened due to its greater acidity and susceptibility to UV rays. Therefore, recently Kim *et al.* tried to use lower-acidic PANI:PSS as the HTL of IOPV.^44,46^ They observed that the thin film formed by PANI:PSS could exhibit a very high transmission level and had a work function value around 5.15 eV. Furthermore, given its lower-acidic nature, the OPV based on PANI:PSS HTL could have a much longer lifespan than a PEDOT:PSS HTL-based IOPVs^44^ (Fig. 9a–c).

Furthermore, Kim *et al.* recently discovered that the performance of an IOPVs (PANI:PSS HTL and P3HT:ICBA active layer) is strongly influenced by the doping concentration of its HTL.^46^ The PANI:PSS HTL might have a better conductivity at an optimal doping concentration. Because of the HTL's improved charge transport capacity, the OPV could achieve an extraordinarily high PCE (8.1%) under the irradiation of a 500 lx LED lamp (having an optimal concentration of the doping reagent) (Fig. 9d–f).

Besides the organic semiconductors, an inorganic semiconductor was also tested as the HTL of IOPVs. In 2020, Shim *et al.* studied the potential of tungsten oxide (WO_3_) as the HTL of an IOPVs.^48^ They considered WO_3_ as an HTL owing to its high work function (≈5.5 eV), low electron affinity, and excellent thermal stability. They discovered that the IOPVs (WO_3_ HTL-based) could show a very high PCE (13%) under the irradiation of a 1000 lx LED due to the excellent hole selectivity (caused by the decreased electron affinity) of the WO_3_ layer (Fig. 9g–i).

### 5.3. Optimization of device architecture through optical simulation

It is an accepted fact that the performance level of a PV device is strongly dependent on various device parameters such as the thickness of various layers, surface morphology of each layer, and absorption coefficient of the active layer, charge carrier mobility, energy band gap, recombination coefficient, and contact resistance between various layers. Therefore, in the past few years, some researchers have tried to optimize these device parameters to achieve maximum PCE.^56,57,59^ It is clear that a purely experimental approach is not very efficient for optimizing device architecture to achieve a better performance level because it will be time-consuming and expensive. Therefore, an alternative approach is needed to address this concern. A combination of experiments and device simulation studies can be a good option, as it can save time and money. Device modeling and simulation can help to explore the device operation mechanism and interaction between various layers of PV devices based on the basic concepts of physics. This is also a very useful and cost-effective tool to find undiscovered device concepts for further development. For the last few years, some people have tried to optimize various parameters of IOPV devices *via* modeling and device simulation studies to achieve better performance.^56,57^ Through meticulous review studies, researchers embarked on a quest to comprehend the intricate interplay of diverse factors shaping the overall performance dynamics of IOPV devices. To elucidate their operational mechanisms, they turned to Finite-Difference Time-Domain (FDTD) simulations. These investigations were underpinned by the utilization of the potent Lumerical FDTD software, renowned for its prowess in optical modeling of photovoltaic systems. In this pursuit, the simulation process commences by ascertaining the optical attributes of the various layers constituting the photovoltaic device. This is achieved through the incorporation of experimentally derived frequency-dependent complex refractive index values for these layers. The mesh density is then tuned to reduce inaccuracy in the findings. The periodic boundaries are then established along the vertical direction of the device axis. Following that, completely matched layers are typically placed alongside the PV axis. The program determines the distribution of normalized electric field intensity within the semiconductor device in the simulation and predicts the amount of absorbed power and optimum short-circuit current density value of the PV device while assuming 100% internal quantum efficiency (because in an optical simulation, the effect of recombination cannot be considered).

As noted earlier, the dimension of the active layer within a PV device holds a pivotal role in shaping the device's PCE. In light of this, endeavors have been undertaken to fine-tune the active layer thickness in various IOPV configurations, with the aim of elevating the PCE. Vincent *et al.* have illuminated this facet through an optical simulation exploration, where they spotlight the potential of a conventional PV structure comprised of P3HT:ICBA. Moreover, their findings advocate for the augmentation of PCE in active-material-based IOPVs employing P3HT:ICBA by judiciously modulating the device's photon absorption capabilities.^59^

**Fig. 10:**
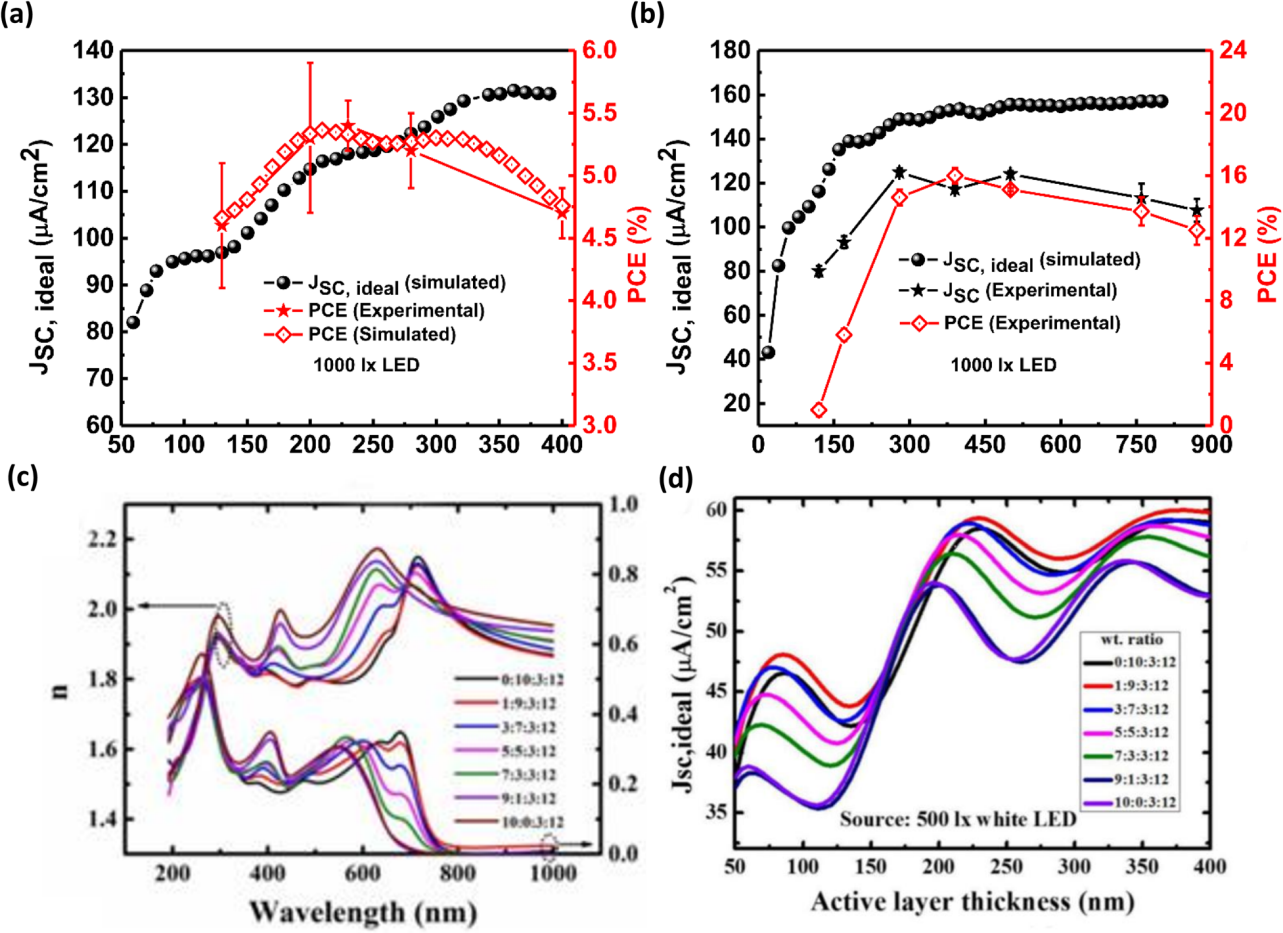
(a) Variation of *J*_SC,ideal_ (simulated), PCE (simulated), and PCE (experimental), value of a P3HT:ICBA active-material-based indoor OPV with active layer thickness; [reproduced with permission.^56^ Copyright 2018, Elsevier]. (b) Variation of *J*_SC,ideal_ (simulated), *J*_SC_ (experimental), and PCE (experimental), value of a PPDT2FBT:PC_70_BM active-material-based indoor OPV with active layer thickness; [reproduced with permission.^51^ Copyright 2019, Elsevier]. (c) Refractive indices (*n*) and the extinction coefficients (*k*) of the different BHJs (PCDTBT:PTB7:PC_61_BM:PC_71_BM) having different composition ratios, (d) *J*_SC,ideal_ (simulated) of different OPVs (having active PCDTBT:PTB7:PC_61_BM:PC_71_BM layer of different composition ratios) under 500 lx white LED illumination; [reproduced with permission.^52^ Copyright 2019, Elsevier].

Later, the same group assessed the suitability of P3HT:ICBA active-material-based indoor OPVs for harvesting low-intensity LED light. They tuned the thickness of the device's active layer using FDTD simulations for different incident light luminance values. They also demonstrated that with an optimal construction, the device may reach maximum PCE. They attempted to develop a technique for improving the PV device architecture to maximize device efficiency using a combination of optical modeling and experimentation^56^ (Fig. 10a).

Using the same method, the impact of active-layer thickness on the level of performance of several semiconductor-based IOPVs was also examined. Shin *et al.*, for example, found that the rate of decrease in ideal short-circuits density value (thus, the PCE value) of a PPDT2FBT:PC_70_BM active-material-based OPVs with increasing active-layer thickness is very slow for indoor applications, whereas the opposite behavior was observed under the 1 sun condition. They have suggested that the effect of critical parasitic resistance on the performance level of an OPV can be varied by changing the illuminating agent^64^ (Fig. 10b).

FDTD optical simulations have been used to optimize the thickness of additional layers, as well as various other characteristics of IOPVs, in addition to the thickness of the active layer. Recently, Lee *et al.* attempted to develop an extremely efficient and adaptable IOPVs device using a quasi-amorphous transparent electrode (ZnO/Ag/ZnO) and P3HT:ICBA active material. Using an FDTD optical simulation analysis, they adjusted thickness of several components of a mechanically stable electrode of an IOPV device. They achieved a higher PCE (12.1%) for the lighting of a 500 lx LED lamp using a mix of modeling and experimental investigation.^55^

FDTD optical simulation studies have also been utilized for optimizing various parameters of quaternary IOPV devices. Shin *et al.* successfully improved the photo-energy absorption ability of the active layer (PCDTBT:PTB7:PC_61_BM:PC_71_BM) of quaternary IOPV devices through the optimization of the weight ratio of various components of the quaternary mixture. They also studied the influence of active-layer thickness on the device's optimal short-circuit current density while working under a 500 lx white LED bulb in order to find the best active-layer thickness^65^ (Fig. 10c and d).

**Fig. 11:**
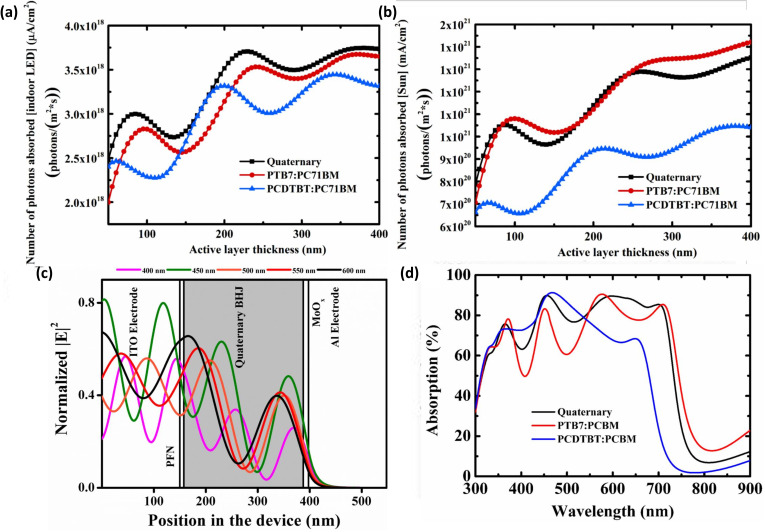
(a) Number of photons absorbed (directly related to the *J*_SC,ideal_) in quaternary and single BHJ photovoltaic cells illuminated with LEDs, (b) number of photons absorbed in quaternary and single BHJ photovoltaic cells illuminated with AM1.5 G, (c) simulated electric field intensity distribution inside the solar structure. The active layer thickness was 230 nm, which resulted in peak absorption under 500 lx indoor white LED illuminations, (d) absorption of quaternary and single BHJ active layers at 230 nm thickness; [reproduced with permission.^60^ Copyright 2019, MDPI].

Vincent *et al.* conducted a comprehensive optical simulation investigation on a PCDTBT:PTB7:PC_61_BM:PC_71_BM active-material-based IOPV device to identify the exact function of the quaternary combination during device operation. They discovered that the thickness (active layer) dependent optimum short-circuit current density value of a quaternary IOPV may oscillate more. Furthermore, because to interference between incoming and reflected (from the back electrode) light, the simulated electric field intensity value had a very high sensitivity within its active to its thickness. They also determined that PCDTBT is the device's primary light absorber, while PTB7 functions as a cascade energy level and a supplemental light absorber during operation^60^ (Fig. 11).

**Fig. 12:**
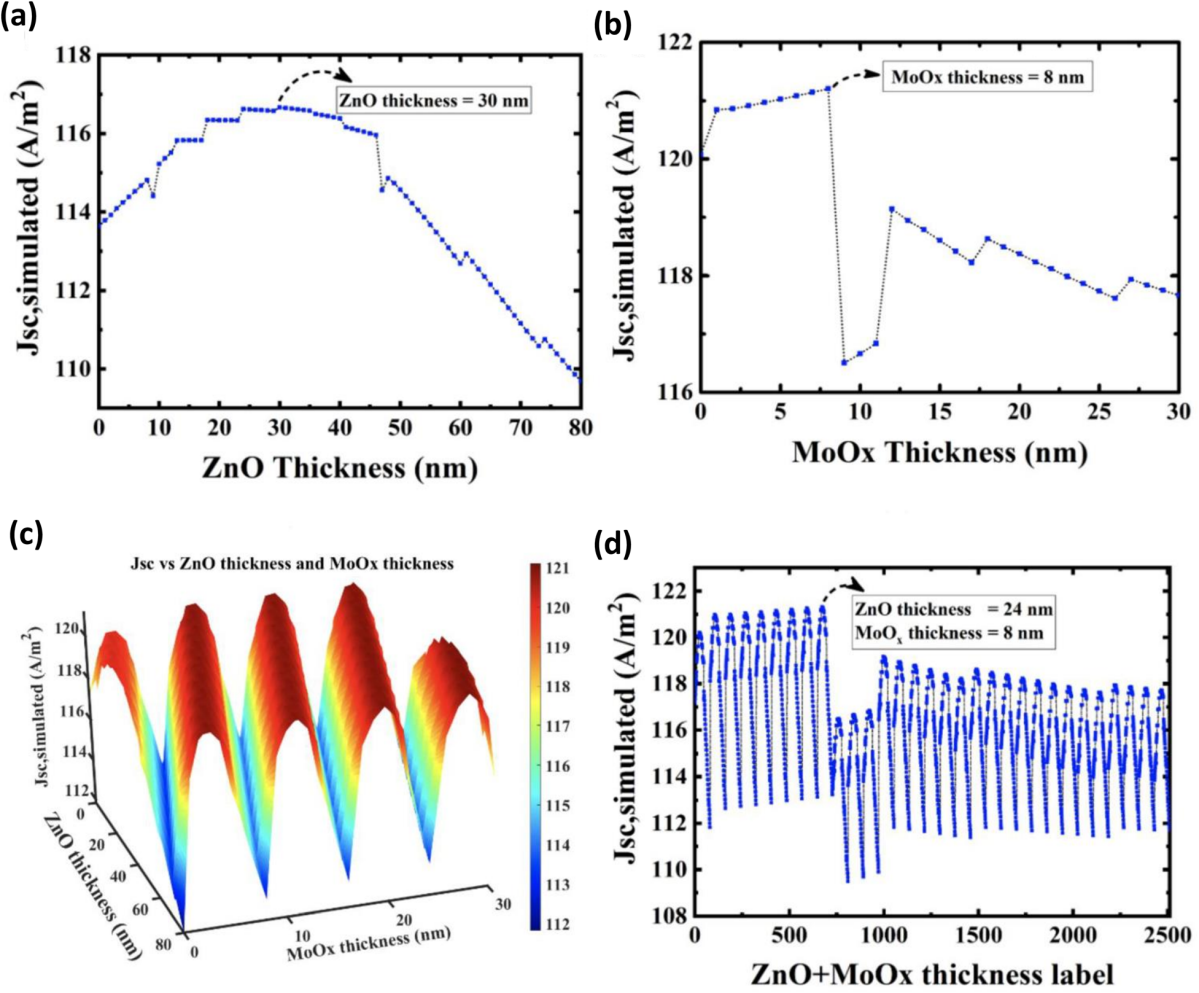
Results of the Brute-force method: (a) single ZnO layer optimization, (b) single MoOx layer optimization, (c) multiple ZnO and MoOx layers optimization, (d) 2D data representation of (c) using labels pointing to the ZnO and MoOx layer thickness combinations for ease of calculation [reproduced with permission.^62^ Copyright 2019, MDPI].

Through an in-depth review study, it becomes evident that achieving the utmost PCE from a PV device is intrinsically linked to the meticulous optimization of the respective layer thicknesses within its structure. In the realm of optimizing these dimensions, the parameter sweep strategy stands as a conventional practice within the Lumerical FDTD solutions software. However, it has become apparent that a brute-force approach is often suboptimal, particularly when aiming for an optimal outcome in varied scenarios. In response to this challenge, Vincent *et al.* embarked on an innovative pathway. They harnessed a genetic algorithm for their device simulation investigation, a method grounded in the principles of Darwinian evolution and natural selection. Notably, their exploration unveiled that this novel optimization methodology accelerates the optimization process and augments precision, in contrast to the traditional brute-force parameter sweep technique^62^ (Fig. 12).

Kim *et al.* recently showed that, while precise balanced band gap estimations show tandem photovoltaics to be feasible for 1 sun conditions, single active layer-based photovoltaics can better collect the low-intensity emission spectra of white LED light sources. The current-matching constraint of a tandem photovoltaic structure coupled in series restricts the device's maximum *J*_SC_ and *V*_OC_, and hence its performance for low-intensity light illumination.^58^ They used optical simulation to estimate different device performance characteristics.

In another study Kim and coworkers designed an indoor OPV having PTB7-Th:PC_70_BM active layer and low-acidic and cheaper polypyrrole:polystyrene sulfonate (PPY:PSS) as the hole transport layer (HTL), by optimizing active layer thickness and processing conditions (spin coating speed and doping concentration) of the HTL *via* optical simulations and experiments. The results show that the device having 100 nm thick active layer and a PPY:PSS-based HTL (PPY : PSS; weight ratio between PPY and PSS 1 : 2) coated at 5000 rpm can exhibit a record high PCE value (16.35%) during its operation under 1000 lx LED lamp. In comparison, a commercially available PEDOT:PSS-based OPV can achieve maximum 14.21% PCE under the same conditions.^101^

From the literature survey, it has been observed that in most cases, varied device parameters of indoor PVs have been optimized *via* optical simulation studies, whereas the effects of generation rate, recombination rate, diffusion of charge carrier, and the collection procedure of electrons on the performance of the PV devices has not been explored. Therefore, electrical simulation studies can be a very good option to realize the effect of variation of various device parameters on the performance of an indoor PV device more precisely.^102^ Thus far, various electrical models such as effective medium model and the Monte Carlo model have been used to perform electrical stimulation of various PV structures. Recently, some researchers have tried to estimate the effect of the morphology of various layers,^103^ domain size,^104^ and weight ratio of donor and acceptor^104^ on the charge transport mechanism of various organic solar cells using the Monte Carlo model. A device simulation investigation may be seen as a highly strong technique for improving the architecture of IOPV devices in this review study. Several attempts have been done over the last four years to improve different device characteristics of various IOPVs using optical simulation studies. Although the generation rate, recombination rate, charge carrier diffusion, and electron and hole collection procedure within the PV device all play important roles during device operation, understanding the effect of the aforementioned parameters on the performance level of an IOPV has not been attempted through electrical simulation studies. As a result, in the near future, a combination of experimental, optical, and electrical modeling investigations of IOPV devices may be done in order to build commercially useable, highly efficient, mechanically flexible next-generation IOPV devices.

## 6. Commercial prospects of IOPVs

Various initiatives for the creation of IOPVs have been implemented throughout the previous decade. Unfortunately, the commercialization of IOPVs has not yet been achieved. A very high PCE (over 20% when illuminated by a 500 lx indoor light) and a greater output power density (30 μW cm^−2^) are not the only characteristics to consider when commercializing OPV devices. There are a number of other considerations that must be made. These issues will be addressed in this section.

First, the lifespan of the IOPVs is a critical consideration. IOPVs have the potential to replace traditional tiny batteries across numerous electrical devices networked inside an IoT network, reducing maintenance costs. To satisfy this requirement, IOPVs must exhibit exceptional environmental resilience, assuring device longevity of a decade or more while maintaining a minimum of 80% original efficiency towards the end of this span. Currently, the operating life of an IOPV is just around two years. As a result, further research is required to develop fundamentally stable constituent materials (including donor and acceptor components, hole transport and electron transport layers, and electrodes) adapted specifically for IOPVs, culminating in their successful commercial realization.

While the importance of stability problems may be reduced in indoor applications, it is worth noting that OPV devices continue to perform poorly when compared to their classic inorganic solar cell counterparts. The several elements impacting device stability, including photodegradation and thermal deterioration, must be given careful consideration. These complicated procedures place restrictions on the devices' long-term performance and dependability.^105^

For the commercialization of IOPVs, the large-scale deposition method is highly encouraged. In such a scenario, the spin coating technology, which is generally utilized, is not a good option. Some studies on IOPVs made with blade coating and lot-die coating, which are more suited to industrial operations, have been reported. Further research is needed on the optimization of various factors, such as solubility of the materials and boiling point of the solvents, during the coating of various layers of an IOPV through various large-area-coating technologies because those factors have a noticeable effect on the morphology, thickness uniformity, and packing of the constant materials of the films. For developing a well-behaved IOPV, the aforementioned properties of various films of the device should be controlled properly.

In addition, the production cost of the device should be minimized. In the near future, it is strongly recommended to develop well-behaved low-cost constituent materials as well as a practical, time-saving, and cost-effective large-area device manufacturing method.

Given the application purpose of IOPVs, the development of efficient, flexible, and stretchable IOPV modules is a necessity. Along with flexibility, the device should exhibit stable performance irrespective of the bending number. For example, Li *et al.* have recently designed a novel intrinsic anti-reflection, high-transmission, and stretchable substrate was designed to construct highly efficient stretchable ITO-free (6k-PDMS/PEDOT:PSS transparent substrate) IOPV.^106^ The stretchable substrate has showed a high diffuse transmittance of over 95% and a transmittance haze of 88%, which can be simply duplicated from the surface texture of commercial 3M abrasive papers. Owing to the high EQE and shunt resistance, the devices with the surface-textured ITO-free stretchable substrates has exhibited a high PCE of 20.5% compared to that of the rigid devices (20.8%) under indoor application. On the other hand, Kim and colleagues have recently reported indium zinc tin oxide bottom electrode-based flexible IOPV with remarkably high mechanical stability.^107^ The OPV shows ≈69% of its initial PCE value after 100 000 bending repetitions for 1000 lx LED illumination. From the review it has been observed that, few researchers have attempted the development of flexible and stretchable IOPVs. Furthermore, the devices' PCE values are inferior to those of their rigid equivalents. As a result, extensive research in this field is necessary.

An IoT network includes several wearable devices. Consequently, a biocompatible powering tool (*i.e.*, IOPVs) for those devices is desirable. It will be highly useful to produce eco-friendly and biocompatible component materials for IOPVs. If IOPVs meet the aforementioned conditions, we anticipate they will achieve commercial-scale manufacturing in near future.

Besides the aforementioned issues, another important concern must be resolved. There is a basic difference between outdoor and indoor lighting conditions. Under outdoor (*i.e.*, 1 sun) conditions, the light is incident directly onto the device, whereas indoors, diffused light (reflected from surfaces such as walls and the surfaces of varied articles) illuminates PV devices. Therefore, during testing of indoor OPVs, the measuring unit must be customized accordingly.^67^ At present, there is no standard protocol for measuring OPVs under indoor lighting conditions. As accurate testing of a PV device is always needed for its commercialization, a standardized measurement system is essential.

## 7. Conclusions

In this systematic review study, it has been found that indoor light energy may be harvested and used to power different tiny and low-powered indoor electronic devices using IOPVs. IOPVs are energy harvesters with unique optoelectronic features and a broad range of applications. Only a few years ago, research into the development of OPVs for indoor applications began. Through the deployment of various opto-electronics methods, IOPVs have already reached above 30% PCE. This value can be further improved; theoretically, it has been calculated that an ideal OPV with an active layer having an energy band gap value within 1.90–2.20 eV can exhibit over 50% PCE under the illumination of FL lamps and LEDs.^16^ However, to achieve this, several issues must be resolved. For example, depending on the light source, the optical energy band gap of photoactive materials must be modified. To decrease energy loss under low-intensity indoor lighting, sufficiently high *V*_OC_-generating active materials must be produced. Furthermore, the optimization of the OPV devices for low-intensity indoor light may differ from the optimization under 1 sun conditions. Although many ETLs have been developed for the enhancement of electron extraction ability of an indoor OPV, there are very few reports on the development of new HTLs for IOPVs for improving their hole extraction efficiency and lifespan. Further research is also needed on developing flexible, large area, and semi-transparent OPVs for their commercialization. Furthermore, currently there is no standardized measurement condition for IOPVs. Different research groups are using different illuminating agents, irradiance power intensity, and luminance value. This makes comparing and evaluating the experimental outcomes challenging. As a result, a consistent measuring procedure for characterizing OPVs under the irradiation of diverse indoor light sources is required.

## Author contributions

The authors confirm contribution to the paper as follows: study conception and design: SB, YJ, and HJ; reference collection: SB, YJ, HJ, and HWL; analysis and interpretation of results SB, YJ, HJ, and HWL; draft manuscript preparation: SB and HK. All authors reviewed the results and approved the final version of the manuscript.

## Conflicts of interest

There are no conflicts to declare.

## Supplementary Material
